# Blood bacterial resistant investigation collaborative system (BRICS) report: a national surveillance in China from 2014 to 2019

**DOI:** 10.1186/s13756-022-01055-5

**Published:** 2022-01-24

**Authors:** Yunbo Chen, Jinru Ji, Chaoqun Ying, Zhiying Liu, Qing Yang, Haishen Kong, Yonghong Xiao, Blood Bacterial Resistant Investigation Collaborative System (BRICS) Study Group, Hui Ding, Yongyun Liu, Haifeng Mao, Ying Huang, Zhenghai Yang, Yuanyuan Dai, Guolin Liao, Lisha Zhu, Liping Zhang, Yanhong Li, Hongyun Xu, Junmin Cao, Baohua Zhang, Liang Guo, Haixin Dong, Shuyan Hu, Sijin Man, Lu Wang, Zhixiang Liao, Rong Xu, Dan Liu, Yan Jin, Yizheng Zhou, Yiqun Liao, Fenghong Chen, Beiqing Gu, Jiliang Wang, Jinhua Liang, Lin Zheng, Aiyun Li, Jilu Shen, Yinqiao Dong, Lixia Zhang, Hongxia Hu, Bo Quan, Wencheng Zhu, Kunpeng Liang, Qiang Liu, Shifu Wang, Xiaoping Yan, Jiangbang Kang, Xiusan Xia, Lan Ma, Li Sun, Liang Luan, Jianzhong Wang, Zhuo Li, Dengyan Qiao, Lin Zhang, Chuandan Wan, Xiaoyan Qi, Fei Du

**Affiliations:** 1 State Key Laboratory for Diagnosis and Treatment of Infectious Diseases, The First Affiliated Hospital, Zhejiang University School of Medicine, Hangzhou, 310003 China; 2 Collaborative Innovation Center for Diagnosis and Treatment of Infectious Diseases, Hangzhou, China; 3 Lishui City Central Hospital, Lishui, China; 4 Affiliated Hospital of Binzhou Medical College, Binzhou, China; 5 The First People’s Hospital of Lianyungang, Lianyungang, China; 6 First Affiliated Hospital of Anhui Medical University, Hefei, China; 7 Yijishan Hospital of Wannan Medical College, Wuhu city, China; 8 Anhui Provincial Hospital, Hefei, China; 9 Wuhan Puren Hospital, Wuhuan, China; 10 The First People’s Hospital of Jingzhou, Jingzhou, China; 11 People’s Hospital of Ningxia Hui Autonomous Region, Yinchuan, China; 12 Anyang District Hospital of Henan Province, Henan, China; 13 The Second People’s Hospital of Yunnan Province, Kunming, China; 14 Zhejiang Provincial Hospital of Traditional Chinese Medicine, Hangzhou, China; 15 People’s Hospital of Huangshan City, Huangshan, China; 16 Mindong Hospital of Ningde City, Ningde, China; 17 The Affiliated Hospital of Jining Medical University, Jining, China; 18 People’s Hospital of Qingyang, Qingyang, China; 19 Tengzhou Centre People’s Hospital, Tengzhou, China; 20 Lu’an People’s Hospital, Hefei, China; 21 Xinjiang Uygur Autonomous Region Youyi Hospital, Ürümqi, China; 22 People’s Hospital of Yichun City, Yichun, China; 23 Jiujiang First People’s Hospital, Jiujiang, China; 24 Shandong Provincial Hospital, Shandong, China; 25 Jingzhou Central Hospital, Jingzhou, China; 26 The First Affiliated Hospital of Gannan Medical University, Ganzhou, China; 27 The First Hospital of Putian City, Putian, China; 28 People’s Hospital of Haining City, Haining, China; 29 Shengli Oilfield Central Hospital, Dongying, China; 30 The Affiliated Hongqi Hospital of Mudanjiang Medicine College, Mudanjiang, China; 31 The Affiliated Hospital of Ningbo Medical School, Ningbo, China; 32 Women’s Hospital, Zhejiang University School of Medicine, Hangzhou, China; 33 The Fourth Affiliated Hospital of Anhui Medical University, Hefei, China; 34 Tianchang City People’s Hospital, Tianchang, China; 35 Shanxi Provincial People’s Hospital, Shanxi, China; 36 The First Affiliated Hospital of Henan University of Science and Technology, Luoyang, China; 37 The Second People’s Hospital of Jingzhou, Honghu, China; 38 Lu’an Civily Hospital, Beijing, China; 39 The Second Affiliated Hospital of Bengbu Medicine College, Bengbu, China; 40 Huaihe Hospital of Henan University, Kaifeng, China; 41 Qilu Children’s Hospital of Shandong University, Jinan, China; 42 Zigong Third People’s Hospital, Zigong, China; 43 The Second Hospital of Shanxi Medical University, Jinzhong, China; 44 The People’s Hospital of Lujiang, Lujiang, China; 45 The First People’s Hospital of Jiayuguan, Jiayuguan, China; 46 The Third Hospital of Hefei, Hefei, China; 47 General Hospital of Northern Theater Command, Shenyang, China; 48 Xingang Hospital of Xinyu, Xinyu, China; 49 The First Affiliated Hospital of Xi’an Medical University, Xi’an, China; 50 Gansu Provincial Hospital of Traditional Chinese Medicine, Lanzhou, China; 51 First People’s Hospital of Chenzhou, Chenzhou, China; 52 Changshu Medicine Examination Institute, Changshu, China; 53 Women and Children’s Hospital of Jin’an District, Fuzhou, China; 54 Hubin Hospital of Hefei, Hefei, China

**Keywords:** Resistance, Bacterial, Bloodstream infection, National resistance surveillance

## Abstract

**Background:**

In this first national bloodstream infection (BSI) surveillance program in China, we assessed the composition of pathogenic bacteria and the trends for antimicrobial susceptibility over a 6-year period in China.

**Methods:**

Blood bacterial isolates from patients at hospitals participating in the Blood Bacterial Resistant Investigation Collaborative System (BRICS) were collected from January 2014 to December 2019. Only the first isolate of a species per patient was eligible over the full study period. Antibiotic-susceptibility testing was conducted by agar-dilution or broth-dilution methods as recommended by the Clinical and Laboratory Standards Institute (CLSI). WHONET 5.6 was used to analyze data.

**Results:**

During the study period, 27,899 bacterial strains were collected. Gram-positive organisms accounted for 29.5% (8244) of the species identified and Gram-negative organisms accounted for 70.5% (19,655). The most-commonly isolated organisms in blood cultures were *Escherichia coli*, *Klebsiella pneumoniae*, *Staphylococcus aureus*, coagulase-negative *Staphylococci*, and *Acinetobacter baumannii*. The prevalence of multidrug-resistant organisms, such as *E. coli*, *K. pneumoniae*, *A. baumannii* was higher in tertiary hospitals, whereas extended-spectrum, β-lactamase-producing *E. coli* (ESBL-*E. coli*), carbapenem-resistant *A. baumannii* were more prevalent in economically-developing areas. The prevalence of methicillin-resistant *S. aureus* declined from 39.0% (73/187) in 2014 to 25.9% (230/889) in 2019 (*p* < 0.05). The prevalence of ESBL-*E. coli* dropped from 61.2% (412/673) to 51.0% (1878/3,683) over time (*p* < 0.05), and carbapenem-resistant *E. coli* remained low prevalence (< 2%; 145/9944; *p* = 0.397). In contrast, carbapenem-resistant *K. pneumoniae* increased markedly from 7.0% (16/229) in 2014 to 19.6% (325/1,655) in 2019 (*p* < 0.05).

**Conclusion:**

*E. coli* and *K. pneumoniae* were the leading causes of BSI during the 6-year study period. The major resistant pathogens declined or remained stable, whereas carbapenem-resistant *K. pneumoniae* continued to increase, which poses a great therapeutic challenge for BSIs.

**Supplementary Information:**

The online version contains supplementary material available at 10.1186/s13756-022-01055-5.

## Background

Antimicrobial resistance (AMR) has become a serious public health threat across the world. AMR control is a priority for the World Health Organization (WHO) [1]. According to WHO recommendations, AMR surveillance is an important part of the AMR control strategy. Furthermore, the data regarding microbiological composition and AMR profiles will guide antimicrobial prescriptions [2]. However, almost all bacterial resistance surveillances programs in China are laboratory-based surveillance strategies with potential biases resulting from optional sample collection. Therefore, it is imperative to carry out infection-defined surveillance to overcome this limitation.

Bloodstream infection (BSI) is a growing public health concern worldwide, with high mortality [3, 4]. It was estimated that the BSI incidence ranged between 113 and 204 per 100,000 in the population [5]. Inappropriate antibiotic therapy for BSI was independently associated with increased risk of mortality [6]. Microbiological epidemiology and bacterial resistance data on BSI will provide a reference for the best empirical antimicrobial therapy [7]. In the face of increasing AMR, precise surveillance has become important in defining the species distribution and resistance of pathogens causing BSI, and thus provide the basis for appropriate empirical therapy.

To comprehensively understand and accurately analyze the microbiological epidemiology and resistance profiles of BSI in China, we initiated the Blood Bacterial Resistant Investigation Collaborative System (BRICS) program in 2014. As an infection-defined surveillance initiative, information on the pathogen distribution and AMR of BSI bacteria was collected from participating hospitals covering 18 provinces in mainland China.

## Material and methods

### Study period and setting

A total of 52 hospitals (100,712 beds), which included 23 tertiary hospitals and 29 non-tertiary hospitals covering 18 provinces in mainland China during 2014–2019, participated in the BRICS program. All participating hospitals were equipped with a qualified microbiology laboratory and followed standardized operational programs. All laboratories participated in one external quality control program at least yearly by either the National Center for Clinical Laboratories or the local province center for clinical laboratories.

### Bacterial isolate collection

Only strains isolated from blood were collected. Blood culture results of patients with only skin contaminants were considered contamination, and patients with both BSI and contamination were classified as having BSI [8]. Coagulase-negative *Staphylococci*, *Bacillus* species, *viridans group Streptococci*, *Corynebacterium* species, *Propionibacterium* species, *Aerococcus* species, and *Micrococcus* species from a single positive culture were excluded as contaminants, while all *Brucella* species were excluded due to the Biosafety Law of China. Only the first isolate of a species per patient was eligible over the full study period. All participating hospitals transferred their strains to the central laboratory quarterly. The central laboratory confirmed the identity of the isolates received using matrix-assisted laser desorption/ionization time of flight mass spectrometry (Bruker Diagnostics, Bremen, Germany) and stored the strains in Microbank® tubes at − 80 °C.

### Antimicrobial susceptibility testing

The antibiotic susceptibilities (minimum inhibitory concentration, MIC) of clinical isolates were determined by agar-dilution or broth-dilution methods [9] at the central laboratory. Extended-spectrum β-lactamase (ESBL) production in *Escherichia coli*, *Klebsiella pneumonia*, and *Proteus mirabilis* using disk diffusion, and inducible clindamycin resistance in *Staphylococcus* species, *Streptococcus pneumonia*, and *Streptococcus* spp. β-hemolytic group were determined. β-hemolytic group determination using disk diffusion (D-zone test) was performed according to the Clinical and Laboratory Standards Institute (CLSI) [9]. The results of MICs were interpreted according to CLSI criteria or European Committee on Antimicrobial Susceptibility Testing (EUCAST) (https://www.eucast.org). *Staphylococcus aureus* ATCC 29,213, *Enterococcus faecalis* ATCC 29,212, *S. pneumonia* ATCC 700,603, *E. coli* ATCC 25,922, *K. pneumoniae* ATCC 27,853, and *Pseudomonas aeruginosa* ATCC 27,853 were included as quality controls.

Polymicrobial bacteremia is defined as a bacteremic episode due to at least two different organisms isolated from the same blood sample, while monomicrobial bacteremia is defined as only one organism isolated in the blood sample [10]. Contaminants are defined as a growth of bacteria in the blood culture bottle that were not present in the patient’s bloodstream but were introduced during sample collection [11]. For surveillance purpose, carbapenem-resistant Enterobacteriaeceae was defined as demonstrating resistance based upon antimicrobial susceptibility test results to at least one of the following carbapenems: ertapenem, meropenem, or imipenem according to the Centers for Disease Control and Prevention (CDC) of USA [12]. Multidrug-resistant (MDR) is defined as non-susceptibility to at least one agent in three or more antimicrobial categories and pan-drug-resistant (PDR) is defined as resistance to all antibiotic classes available for empirical treatment [13].

### Data and statistical analysis

To compare resistance profiles in different economic areas, a developed or developing area was defined as having a per capita gross domestic product (GDP) ≥ 11,000 USD or < 11,000 USD in 2019. WHONET 5.6 was used to analyze the distribution and resistance rates of the isolates. To assess trends in proportions of resistant isolates over time, the Cochran–Armitage χ^2^ test was used. The Chi-square test was used to compare rates for different hospital levels and regions. Significance was assumed at a *p*-value < 0.05. Statistical analysis was performed using SPPS (version 9).

### Ethics requirements

For this observational study, the need for patient consent was not required. Data were not identifiable back to the patients from whom they originated; an ethics approval was waived.

## Results

### Bacterial isolate collection

A total of 52 hospitals from 2014 to 2019 participated in BRICS. There were 8 tertiary hospitals and 13 non-tertiary hospitals that participated over the entire duration of the surveillance program, while other hospitals participated for 1 to 5 years. The number of participating hospitals per year were as follows: 21 in 2014, 26 in 2015, 27 in 2016, 30 in 2017, 39 in 2018, and 49 in 2019.

**Fig. 1:**
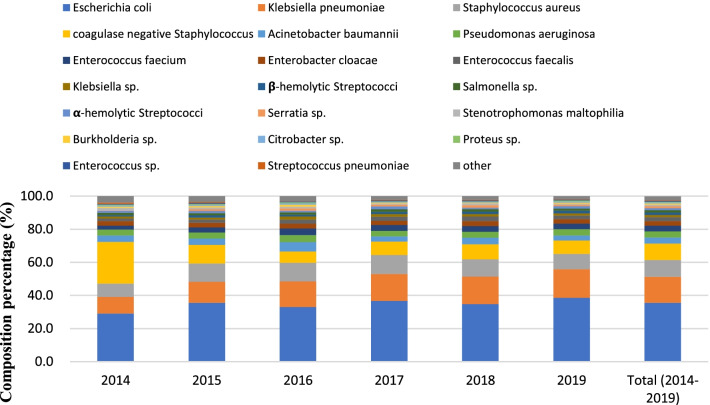
The bacterial spectrum of bloodstream infections by year (relative proportions) from 2014 to 2019

Over the 6-year period, 27,899 isolates were collected in total; Gram-positive organisms (GPO) accounted for 29.5% (8244/27,899) and Gram-negative organisms (GNO) for 70.5% (19,655/27,899). GNO increased from 58.4% in 2014 to 73.0% in 2019. *E. coli* and *K. pneumoniae* were the most common organisms overall, followed by *S. aureus*, coagulase-negative *Staphylococci* (CoNS), *Acinetobacter baumannii*, and *P. aeruginosa* (Fig. 1). Notably, *E. coli* increased 29.1% to 38.5% during the study period. A higher proportion of CoNS were detected in tertiary hospitals and developing areas (10.1% and 12.1%, respectively) than those in non-tertiary hospitals or developed areas (9.4% and 7.7%, respectively). The percentage of *E. coli* was higher in non-tertiary hospitals (38.8%) than in tertiary hospitals (32.5%). *P. aeruginosa* and *A. baumannii* were more often detected in tertiary hospitals (Additional file 1: Table S1).

### Bacterial resistance and trends over time

#### Resistance in Gram-positive organisms

**Fig. 2:**
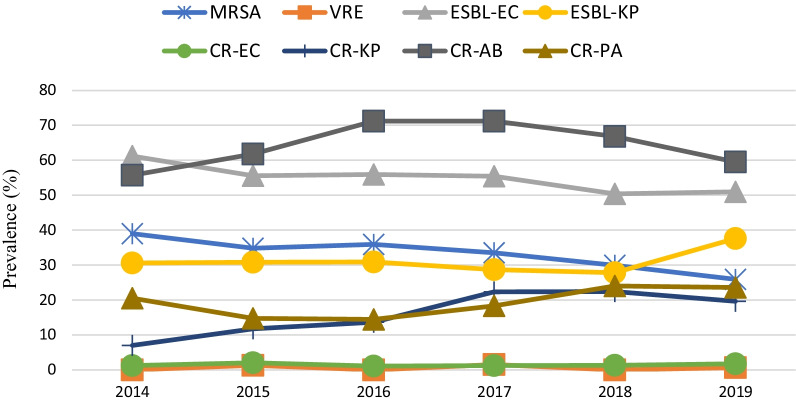
Trends in the prevalence of major drug-resistant bacteria over the study period. CR-AB, carbapenem-resistant *A. baumannii*; CR-EC, carbapenem-resistant *E. coli*; CR-KP, carbapenem-resistant *K. pneumoniae*; CR-PA, carbapenem-resistant *P. aeruginosa*; ESBL-EC, extended-spectrum β-lactamase-producing *E. coli*; ESBL-KP, extended-spectrum β-lactamase-producing *K. pneumoniae*; MRSA, methicillin-resistant *S. aureus*; VRE, vancomycin-resistant *E. faecium*

The overall prevalence of methicillin-resistant *Staphylococcus aureus* (MRSA) declined during the study period from 39.0% to 25.9% (*p* < 0.05) (Table 1, Fig. 2). No vancomycin resistance, or “MIC creep” was detected over time. Of note, the prevalence of MRSA was higher in developed regions (33.9% vs 28.3% in developing regions), but no differences across different hospital levels was observed (Additional file 1: Table S2). Moreover, we observed a decline in the proportion of rifampicin-resistant *S. aureus* during the study period (5.3% in 2014 and 1.3% in 2019, *p* < 0.05).

**Table 1:** The susceptibility and resistance trends of Gram-positive pathogens to antimicrobial agents

	All strains (204–2019)					2014		2015		2016	
	Number	MIC_50_	MIC_90_	%R	%S	%R	%S	%R	%S	%R	%S
*S. aureus*											
MDR	2843	–	–	54.9	–	55.6	–	55.1	–	60.9	–
penicillin G	2667	0.5	16	87.9	12.1	91.2	8.8	88.1	11.9	84.2	15.8
oxacillin	2843	0.5	32	31.3	68.7	39.0	61.0	34.8	65.2	35.9	64.1
amikacin	2843	4	8	3.7	91.6	3.2	86.6	5.1	90.2	4.9	92.1
gentamicin	2360	0.5	32	15.3	84.1	–	–	–	–	17.4	81.7
rifampicin	2843	0.016	0.016	3.0	96.6	5.3	94.1	3.0	95.9	4.1	95.3
ciprofloxacin	2360	0.5	16	21.3	74.6	–	–	–	–	23.9	71.4
levofloxacin	2843	0.25	16	17.9	80.7	21.4	76.5	19.6	78.7	16.2	81.3
moxifloxacin	2843	0.125	4	17.6	78.8	23.5	68.4	20.3	62.8	17.9	78.5
trimethoprim/sulfamethoxazole	2843	0.064	0.25	2.8	97.2	4.8	95.2	7.1	92.9	3.9	96.1
clindamycin	2843	0.25	32	37.0	60.4	29.9	67.4	35.8	61.1	33.1	60.4
erythromycin	2843	32	32	60.1	31.5	64.7	33.7	65.5	28.0	63.3	27.4
daptomycin	2843	0.5	1	0	98.9	0	97.9	0	98.3	0	97.2
linezolid	2843	1	2	0	100	0	100	0	100	0	100
vancomycin	2843	1	2	0	100	0	100	0	100	0	100
teicoplanin	2360	0.5	2	0	100	–	–	–	–	0	100
tetracycline	2843	0.25	32	19.4	77.2	23.5	72.7	24.0	75.7	25.0	70.6
tigecycline	2843	0.125	0.25	1.2	98.8	1.1	98.9	0.7	99.3	4.3	95.7
*E. faecium*											
MDR	950	–	–	92.7	–	85.7	–	81.0	–	91.2	–
penicillin G	950	32	64	88.8	11.2	87.5	12.5	87.3	12.7	86.7	13.3
ampicillin	950	32	32	86.8	13.2	82.1	17.9	88.6	11.4	83.4	16.6
rifampicin	815	8	8	82.2	11.3	–	–	–	–	74.6	15.5
ciprofloxacin	815	16	32	91.9	6.0	–	–	–	–	86.7	8.8
levofloxacin	950	16	32	86.3	8.6	96.4	1.8	77.2	13.9	84.0	11.6
erythromycin	950	32	32	87.9	3.9	83.9	1.8	81.0	6.3	87.8	5.0
daptomycin	950	2	4	0.1	99.9	0	100	1.3	98.7	0	100
linezolid	950	2	2	0.5	93.2	0	100	1.3	98.7	0	100
vancomycin	949	1	2	0.5	97.9	0	100	1.3	98.7	0	100
teicoplanin	815	0.25	1	0.2	99.8	–	–	–	–	0	100
tigecycline	950	0.064	0.5	0.3	87.4	0	98.2	0	96.2	1.1	98.9
*E. faecalis*											
MDR	636	–	–	39.2	–	40.0	–	19.2	–	33.6	–
Penicillin G	636	2	8	9.0	91.0	17.1	82.9	9.6	90.4	9.7	90.3
Ampicillin	636	1	4	7.4	92.6	11.4	88.6	0	100	10.6	89.4
Rifampicin	549	4	8	50.1	23.5	–	–	–	–	52.2	11.5
Ciprofloxacin	549	1	16	25.7	57.2	–	–	–	–	21.2	64.6
Levofloxacin	636	2	16	26.1	64.5	37.1	60.0	44.2	55.8	18.6	76.1
Erythromycin	636	32	32	58.3	13.5	68.6	100	51.9	98.1	61.1	18.6
Daptomycin	636	1	2	0.3	98.4	0	95.3	1.9	98.1	0.9	97.3
Linezolid	636	2	2	1.3	98.7	0	100	3.8	96.2	0.9	99.1
Vancomycin	636	1	2	0	99.4	0	100	0	100	0	100
Teicoplanin	549	0.25	0.25	0.2	99.8	–	–	–	–	0	100
Tigecycline	636	0.125	0.25	0.6	95.4	0	97.1	0	65.4	0	100

	2017		2018		2019		Trend	*p*
	%R	%S	%R	%S	%R	%S		
*S. aureus*								
MDR	54.2	–	48.3	–	57.9	–	–	0.590
penicillin G	81.2	18.8	91.1	8.9	90.7	9.3	↓	0.004
oxacillin	33.5	66.5	29.9	70.1	25.9	74.1	↓	0.000
amikacin	6.0	84.3	2.4	92.3	2.2	95.7	↓	0.006
gentamicin	19.5	80.5	15.1	84.0	12.4	87.2	↓	0.002
rifampicin	6.0	94.0	1.5	98.2	1.3	98.2	↓	0.000
ciprofloxacin	22.2	72.3	21.9	77.0	19.0	75.9	–	0.031
levofloxacin	20.2	79.3	21.3	76.3	14.4	85.3	↓	0.050
moxifloxacin	20.2	79.3	18.0	78.9	13.7	86.1	↓	0.000
trimethoprim/sulfamethoxazole	1.0	99.0	3.5	96.5	0.9	99.1	↓	0.000
clindamycin	33.7	62.7	29.1	69.4	47.4	52.0	↑	0.000
erythromycin	62.2	22.7	59.4	32.2	55.0	38.2	↓	0.000
daptomycin	0	98.3	0	100	0	100	–	NA
linezolid	0	100	0	100	0	100	–	0.021
vancomycin	0	100	0	100	0	100	–	NA
teicoplanin	0	100	0	100	0	100	–	NA
tetracycline	23.1	73.7	14.0	79.2	15.4	82.9	↓	0.000
tigecycline	1.2	98.8	0.4	99.6	0.1	99.9	↓	0.000
*E. faecium*								
MDR	95.4	–	98.3	–	93.5	–	↑	0.000
penicillin G	90.8	9.2	89.4	10.6	89.5	10.5	–	0.364
ampicillin	90.0	10.0	87.7	12.3	87.4	12.6	–	0.320
rifampicin	77.7	10.8	89.9	8.9	84.0	10.5	↑	0.002
ciprofloxacin	94.6	3.1	97.2	1.7	90.8	8.0	–	0.214
levofloxacin	92.3	4.6	90.5	2.8	83.4	11.7	–	0.553
erythromycin	84.6	6.9	93.3	4.5	88.6	1.5	–	0.044
daptomycin	0	100	0	100	0	100	–	0.139
linezolid	0.8	99.2	1.1	98.9	0.3	99.7	–	0.931
vancomycin	1.5	98.5	0	92.2	0.6	99.1	–	0.935
teicoplanin	0.8	99.2	0	100	0.3	99.7	–	0.807
tigecycline	0	97.7	0	100	0.3	98.5	–	0.710
*E. faecalis*								
MDR	46.7	–	55.6	–	23.5	–	–	0.815
Penicillin G	12.0	88.0	8.3	91.7	6.0	94.0	↑	0.000
Ampicillin	18.5	81.5	2.1	97.9	5.5	94.5	–	0.081
Rifampicin	44.6	29.3	52.8	13.2	49.5	35.0	–	NA
Ciprofloxacin	31.5	46.7	20.8	69.4	29.0	49.0	–	0.126
Levofloxacin	28.3	69.6	24.3	41.0	24.0	75.5	↓	0.000
Erythromycin	56.5	22.8	61.1	4.9	55.5	8.0	↓	0.010
Daptomycin	0	95.7	0	98.6	0	100	–	0.137
Linezolid	0	100	2.8	97.2	0.5	99.5	–	0.710
Vancomycin	0	100	0	97.2	0	100	–	NA
Teicoplanin	1.1	98.9	0	100	0	100	↓	0.019
Tigecycline	3.3	90.2	0	100	0.5	99.5	↑	0.031

MDR, multidrug-resistance; NA, no account. ↑, resistance trend with increase. ↓, resistance trend with decrease. -, no significant change

The methicillin-resistant, coagulase-negative *Staphylococci* (MRCNS) prevalence was 74.3% and no glycopeptide-resistant MRCNS were detected (Additional file 1: Table S2). Compared with MRSA, MRCNS exhibited higher resistance rates to fluoroquinolones and trimethoprim/sulfamethoxazole (Additional file 1: Table S3).

*E. faecium* showed increased resistance rates to rifampicin over time (*p* < 0.05) (Table 1). Of note, the overall prevalence of vancomycin-resistant *E. faecium* (VRE) was low. Daptomycin- or linezolid-resistant *E. faecium* was rare (≤ 0.1% and ≤ 0.5% overall, respectively). The prevalence of MDR *E. faecium* was similar across different hospital levels and economic regions (Additional file 1: Table S4). 7.4% *E. faecalis* were resistant to ampicillin (Table 1).

*S. pneumoniae* was susceptible to penicillin G as defined by the breaking point of non-meningitis injection of penicillin G (Additional file 1: Table S5). None of the 388 *Streptococcus* spp. β-hemolytic group were resistant to penicillin G, while 1.4% of α-hemolytic *Streptococci* strains was resistant (Additional file 1: Table S2).

#### Resistance of Enterobacteriaceae

Carbapenem-resistant (CR) *E. coli* (CR-*E. coli*) and CR *K. pneumoniae* (CR-*K. pneumoniae*) increased from 1.2% and 7.0%, respectively, in 2014 to 1.7% and 19.6%, respectively, in 2019 (*p* = 0.397 and *p* < 0.05, respectively) (Table 2, Fig. 2). Unlike *E. coli*, MDR *K. pneumoniae* increased significantly over a 6-year period (*p* < 0.05). Overall, the resistance rate of CR-*E. coli* to ceftazidime/avibactam was 60.7% whereas 8.3% CR-*K. pneumoniae* were resistant to ceftazidime/avibactam (Additional file 1: Tables S6 and S7). It is interesting to note that the prevalence of CR-*K. pneumonia* was higher in tertiary hospitals and developed regions compared with non-tertiary hospitals and developing regions (both *p* < 0.05). However, there was no significant difference in the prevalence of CR-*E. coli* between developed and developing regions (Additional file 1: Table S4).

**Table 2:** The susceptibility and resistance trends of Gram-negative pathogens to antimicrobial agents

	All strains (204–2019)					2014		2015		2016	
	Number	MIC_50_	MIC_90_	%R	%S	%R	%S	%R	%S	%R	%S
*E. coli*											
MDR	9944	–	–	71.4	–	76.2	–	65.8	–	72.1	–
ESBL-*E. coli*	9944	–	–	53.4	–	61.2	–	55.6	–	55.9	–
CR-*E. coli*	9944	–	–	1.5	–	1.2	–	2.0	–	1.1	–
amoxicillin	9944	128	256	74.8	20.4	93.2	5.1	83.4	15.8	84.9	14.4
amoxicillin/clavulanic acid	9944	16	256	39.3	40.6	26.9	40.6	14.6	60.7	18.0	56.4
piperacillin/tazobactam	9944	2	64	7.5	86.4	12.3	78.0	8.1	88.3	6.7	89.1
cefoperazone/sulbactam^a^	9944	4	32	8.5	80.6	15.3	65.5	9.3	76.5	8.0	79.5
ceftazidime/avibactam	3683	0.25	1	1.0	99.0	–	–	–	–	–	–
cefazolin	9944	128	128	59.7	34.8	69.1	26.7	62.6	30.7	63.5	29.8
cefuroxime	9944	128	128	55.0	41.5	64.8	31.8	58.7	40.1	59.9	37.4
ceftazidime	9944	1	64	28.9	63.2	33.9	58.7	30.6	62.3	30.9	61.9
ceftriaxone	9944	16	64	55.8	43.8	65.8	33.7	57.8	41.9	59.6	40.3
cefepime	9944	1	32	21.0	58.4	30.2	46.7	24.4	54.1	25.8	51.7
cefoxitin	9944	4	32	13.6	77.6	19.5	69.4	13.5	80.4	13.9	74.6
moxalactam	9943	0.25	2	2.5	95.6	3.0	93.9	2.3	94.4	2.6	94.9
aztreonam	9944	4	64	36.2	55.6	42.2	49.5	37.5	54.8	37.6	53.6
ertapenem	5495	0.016	0.064	1.5	97.9	–	–	–	–	–	–
imipenem	9944	0.125	0.5	1.4	98.3	1.0	97.8	1.4	98.1	1.1	98.7
meropenem	9944	0.016	0.064	1.4	98.5	1.2	98.7	1.5	98.2	1.1	98.7
amikacin	9944	4	8	2.9	96.7	4.9	93.8	3.3	96.3	3.6	95.5
gentamicin	9944	4	128	39.5	58.7	44.0	51.9	39.9	57.1	43.3	53.6
ciprofloxacin	9944	4	32	64.3	26.0	65.7	27.0	58.9	30.9	74.0	17.9
levofloxacin	9944	4	32	53.3	37.1	61.7	31.6	53.1	37.2	57.5	34.3
trimethoprim/sulfamethoxazole	9944	8	8	57.1	42.9	60.8	39.2	58.3	41.7	58.7	41.3
fosfomycin	9944	0.5	4	1.7	95.2	0.1	93.2	0.2	95.5	0.6	94.5
polymyxin B	9944	0.5	2	2.2	97.8	1.5	98.5	1.4	98.6	7.2	92.8
tigecycline	9944	0.25	0.5	0	100	0	99.6	0	100	0	100
*K. pneumoniae*											
MDR	4378	–	–	38.7	–	30.6	–	32.6	–	36.1	–
ESBL-*K. pneumoniae*	4378	–	–	32.5	–	30.6	–	30.8	–	30.9	–
CR-*K. pneumoniae*	4378	–	–	18.3	–	7.0	–	11.7	–	13.6	–
amoxicillin/clavulanic acid	3679	8	128	36.0	52.4	29.7	45.4	21.4	67.7	32.5	56.7
piperacillin/tazobactam	4378	4	128	22.0	70.5	14.0	81.2	15.5	79.5	20.1	75.5
cefoperazone/sulbactam^a^	4378	1	128	24.9	69.7	14.4	76.9	19.4	73.9	22.3	71.5
ceftazidime/avibactam	1655	0.5	4	1.6	98.4	–	–	–	–	–	–
cefazolin	4378	2	128	43.5	52.5	40.6	55.9	40.2	55.1	43.3	50.4
cefuroxime	4378	8	128	42.7	54.7	37.1	61.1	37.5	59.5	43.1	54.7
ceftazidime	4378	0.5	64	31.6	64.9	20.1	73.8	27.0	69.8	30.9	64.8
ceftriaxone	4378	0.125	64	40.2	59.3	34.5	65.5	37.5	62.2	39.5	60.0
cefepime	4378	0.125	64	27	64.8	16.2	72.9	20.8	70.7	26.5	65.5
cefoxitin	4378	4	128	27.3	68.6	22.3	75.5	20.5	73.6	25.5	70.1
moxalactam	4378	0.5	128	17.4	80.3	6.6	92.1	10.0	87.1	14.3	83
aztreonam	4378	0.125	64	34.1	63.7	24.0	72.9	29.0	70.4	32.5	65.5
ertapenem	2518	0.016	32	20.5	78.6	–	–	–	–	–	–
imipenem	4378	0.25	32	17.5	81.6	5.2	93.9	9.7	88.9	13.2	86.0
meropenem	4378	0.032	32	17.4	82.2	5.7	93.4	9.7	89.1	12.6	86.8
amikacin	4378	2	128	13.7	86.2	6.1	93.9	9.1	90.9	12.2	87.5
gentamicin	4378	1	128	26.8	72.4	20.1	78.6	21.1	78.0	26.5	72.6
ciprofloxacin	4378	0.25	32	44.5	51.7	36.7	58.5	40.2	56.3	43.3	50.4
levofloxacin	4378	0.25	32	34.1	55.8	25.3	60.7	26.1	61.9	31.4	58.0
trimethoprim/sulfamethoxazole	4378	0.125	8	36.8	63.2	31.9	68.1	35.8	64.2	32.5	67.5
fosfomycin	4378	4	128	6.1	89.6	0.4	90.8	0.6	90.0	2.9	91.7
polymyxin B	4378	1	2	3.6	96.4	0.9	99.1	1.5	98.5	5.7	94.3
tigecycline	4378	0.5	1	0.2	98.9	0.9	95.2	1.5	97.4	0.1	99.1
*E. cloacae*											
MDR	–	–	–	20.8	–	24.6		28.9		23.8	
piperacillin/tazobactam	785	2	128	13.1	78.5	9.2	84.6	11.8	80.3	14.6	76.9
cefoperazone/sulbactam^a^	785	0.5	32	9.8	80.4	7.7	83.1	15.8	75.0	8.5	76.9
ceftazidime/avibactam	265	0.25	4	6.0	94.0	–	–	–	–	–	–
ceftazidime	785	0.25	64	29.6	67.5	27.7	69.2	23.7	72.4	28.5	69.2
ceftriaxone	785	0.25	64	39.9	59.5	52.3	46.2	42.1	57.9	40.8	59.2
cefepime	785	0.125	16	13.4	78.7	6.2	84.6	13.2	81.6	12.3	75.4
moxalactam	785	0.25	32	6.2	84.2	3.1	86.2	5.3	81.6	6.9	83.1
aztreonam	785	0.125	64	29.7	68.4	27.7	67.7	25.0	75.0	29.2	69.2
ertapenem	423	0.016	0.5	5.4	92.4	–	–	–	–	–	–
imipenem	785	0.25	1	4.8	93.0	1.5	90.8	5.3	92.1	4.6	92.3
meropenem	785	0.032	0.125	4.5	94.6	0	98.5	3.9	94.7	4.6	91.5
amikacin	785	2	4	2.3	97.3	4.6	95.4	1.3	98.7	3.8	95.4
gentamicin	785	1	64	14.5	81.7	12.3	83.1	11.8	77.6	13.8	83.8
ciprofloxacin	785	0.064	16	25.0	69.3	24.6	63.1	26.3	69.7	25.4	67.7
levofloxacin	785	0.064	8	17.7	73.8	23.1	69.2	21.1	69.7	17.7	73.8
trimethoprim/sulfamethoxazole	785	0.125	8	25.1	74.9	41.5	58.5	31.6	68.4	19.2	80.8
fosfomycin	785	8	64	1.1	93.2	0	84.6	0	90.8	0.8	93.8
polymyxin B	785	1	32	28.7	71.3	1.5	98.5	0	100	20.0	80.0
tigecycline	785	0.25	1	0.8	98.2	1.5	98.5	0.5	98.2	2.3	96.9
*Klebsiella* spp											
MDR	401	–	–	20.0	–	44.4		21.9		35.4	
Piperacillin/tazobactam	401	1	128	15.5	79.3	33.3	63.0	9.4	90.6	19.0	78.5
Cefoperazone/Sulbactam^a^	401	4	128	18.0	74.1	14.8	77.8	9.4	84.4	12.7	74.7
Ceftazidime/avibactam	130	0.25	4	4.6	95.4	–	–	–	–	–	–
Cefazolin	198	4	128	43.9	37.9	72.7	18.2	40.9	45.5	60.5	32.6
Cefuroxime	266	8	128	38.0	58.3	59.3	37.0	31.2	62.5	62.8	34.9
Ceftazidime	401	0.5	64	28.7	68.3	37.0	48.1	18.8	81.2	32.9	64.6
Ceftriaxone	401	0.25	64	39.4	58.9	63.0	37.0	25.0	75.0	50.6	48.1
Cefepime	401	0.125	16	15.0	79.1	18.5	63.0	12.5	81.2	22.8	68.4
Cefoxitin	198	4	128	18.2	76.8	54.5	45.5	18.2	81.8	16.3	76.7
Moxalactam	400	0.25	16	7.5	88.5	7.4	88.9	3.1	93.8	6.3	89.9
Aztreonam	401	0.25	64	28.7	67.8	51.9	37.0	21.9	78.1	31.6	62.0
Ertapenem	204	0.016	1	7.4	89.2	–	–	–	–	–	–
Imipenem	401	0.25	2	6.2	89.8	3.7	88.9	3.1	96.9	5.1	89.9
Meropenem	401	0.032	0.25	5.0	94.0	0	100	3.1	96.9	3.8	93.7
Amikacin	401	2	8	2.5	97.3	7.4	92.6	3.1	96.9	3.8	94.9
Gentamicin	401	1	64	12.0	86.0	14.8	85.2	12.5	78.1	26.6	70.9
Ciprofloxacin	401	0.064	32	30.2	67.3	48.1	48.1	31.2	68.8	49.4	49.4
Levofloxacin	401	0.125	8	21.2	72.1	44.4	51.9	21.9	68.8	30.4	58.2
Trimethoprim/Sulfamethoxazole	401	0.125	8	19.0	81.0	37.0	63.0	31.2	68.8	27.8	72.2
Fosfomycin	401	4	64	2.5	93.5	0	92.6	0	84.4	2.5	93.7
Polymyxin B	401	1	2	6.0	94.0	0	100	0	100	5.1	94.9
Tigecycline	401	0.25	1	1.2	97.8	7.4	92.6	6.2	93.8	1.3	96.2
*Salmonella* spp											
MDR	–	–	–	45.1	–	36.4	–	29.4	–	38.7	–
Amoxicillin	384	128	256	57.6	40.6	57.6	39.4	50.0	50.0	37.1	59.7
Amoxicillin/Clavulanic Acid	384	8	128	7.6	91.6	8.2	57.6	5.9	82.4	4.5	35.5
Piperacillin/Tazobactam	384	1	16	1.6	95.8	0	87.9	5.9	100	1.6	100
Cefoperazone/Sulbactam^a^	384	2	8	2.1	95.8	9.1	90.9	0	94.1	0	98.4
Ceftazidime/Avibactam	117	0.5	0.5	0	100	–	–	–	–	–	–
Ceftazidime	384	0.25	4	9.9	90.1	12.1	87.9	8.8	91.2	4.8	95.2
Ceftriaxone	384	0.125	16	10.9	88.8	15.2	84.8	5.9	91.2	6.5	93.5
Cefepime	384	0.125	1	5.7	91.4	0	90.9	2.9	97.1	6.5	93.5
Moxalactam	384	0.25	8	1.0	95.6	0	100	0	94.1	1.6	93.5
Aztreonam	384	0.125	8	6.2	84.9	12.1	84.8	2.9	97.1	1.6	74.2
Ertapenem	201	0.008	0.016	0.5	99.5	–	–	–	–	–	–
Imipenem	384	0.064	0.25	0.3	99.2	0	100	0	97.1	0	100
Meropenem	384	0.032	0.125	1.6	96.4	3.0	97.0	0	100	1.1	91.9
Amikacin	384	2	8	1.0	98.7	3.0	97.0	0	100	0	100
Gentamicin	384	1	4	7.3	91.9	9.1	90.9	2.9	97.1	6.5	93.5
Ciprofloxacin	384	0.5	1	18	29.7	18.2	36.4	14.7	35.3	25.8	17.7
Levofloxacin	384	0.25	1	8.6	33.1	6.1	33.3	5.9	32.4	3.2	30.6
Trimethoprim/Sulfamethoxazole	384	0.125	8	13.8	86.2	15.2	84.8	11.8	88.2	4.8	95.2
Fosfomycin	384	0.5	4	0.5	99.0	0	97.0	0	100	0	100
Polymyxin B	384	2	16	38.3	61.7	0	100	0	100	37.1	62.9
Tigecycline	384	0.25	1	1.3	97.4	0	100	2.9	97.1	0	91.9
*Serratia* spp											
MDR	–	–	–	9.3	–	37.5	–	10.3	–	11.9	–
Piperacillin/Tazobactam	313	1	64	6.4	87.2	25.0	75.0	6.9	82.8	6.0	85.1
Cefoperazone/Sulbactam^a^	312	1	64	16.0	75.6	25.0	50.0	17.2	72.4	4.5	82.1
Ceftazidime/Avibactam	83	0.125	0.25	0	100	–	–	–	–	–	–
Ceftazidime	311	0.25	8	9.6	89.1	37.5	62.5	6.9	89.7	10.4	88.1
Ceftriaxone	312	0.125	64	28.8	71.2	37.5	62.5	31.0	69.0	25.4	74.6
Cefepime	313	0.125	16	18.5	74.8	25.0	75.0	20.7	72.4	11.9	80.6
Moxalactam	312	0.5	4	3.2	94.9	12.5	87.5	0	100	1.5	94.0
Aztreonam	313	0.125	32	16.0	72.8	25.0	75.0	24.1	75.9	19.4	76.1
Ertapenem	169	0.016	0.064	5.3	94.1	–	–	–	–	–	–
Imipenem	313	0.5	2	7.0	89.1	37.5	62.5	3.4	96.6	3.0	94.0
Meropenem	313	0.064	0.25	5.1	93.0	25.0	75.0	0	96.6	1.5	94.0
Amikacin	313	4	16	1.0	98.4	0	100	0	100	0	97.0
Gentamicin	313	2	128	20.1	79.2	25.0	62.5	20.7	79.3	19.4	79.1
Ciprofloxacin	312	0.25	16	30.4	50.0	25.0	50.0	31.0	69.0	29.9	47.8
Levofloxacin	313	0.5	16	25.9	68.1	25.0	62.5	20.7	75.9	25.4	70.1
Trimethoprim/Sulfamethoxazole	313	0.125	0.5	6.1	93.9	37.5	62.5	10.3	89.7	11.9	88.1
Fosfomycin	312	4	8	0.6	98.7	0	100	0	100	0	100
Tigecycline	312	0.5	2	1.5	97.5	7.5	92.5	1.7	98.3	1.5	98.5

	2017		2018		2019		Trend	*p*
	%R	%S	%R	%S	%R	%S		
*E. coli*								
MDR	71.2	–	71.1	–	71.8	–	–	0.749
ESBL-*E. coli*	55.4	–	50.4	–	51.0	–	↓	0.000
CR-*E. coli*	1.2	–	1.3	–	1.7	–	–	0.397
amoxicillin	85.6	13.9	84.6	14.5	83.4	15.0	↓	0.000
amoxicillin/clavulanic acid	19.7	51.5	24.2	48.7	44.0	38.1	↑	0.000
piperacillin/tazobactam	6.2	89.0	5.6	82.5	8.1	83.0	–	0.04
cefoperazone/sulbactam^a^	8.7	80.7	7.0	91.1	7.8	84.0	↓	0.000
ceftazidime/avibactam	–	–	–	–	1.0	99.0	–	NA
cefazolin	61.8	31.2	57.1	37.0	56.1	39.6	↑	0.000
cefuroxime	50.6	45.4	53.6	43.0	52.7	43.1	↓	0.000
ceftazidime	28.9	62.6	25.6	65.8	28.2	63.7	↓	0.000
ceftriaxone	57.3	42.1	53.5	46.0	52.6	47.2	↓	0.000
cefepime	24.9	52.4	20.6	58.5	15.3	66.6	↓	0.000
cefoxitin	17.0	74.4	9.5	82.9	13.1	78.1	↓	0.000
moxalactam	2.5	95.5	2.3	96.8	2.5	95.9	–	0.661
aztreonam	34.9	54.8	30.9	59.8	37.3	55.9	↓	0.021
ertapenem	–	–	1.3	98.2	1.7	97.7	–	0.281
imipenem	1.2	98.4	1.3	98.5	1.6	98.2	–	0.140
meropenem	1.0	98.6	1.3	98.6	1.6	98.4	–	0.217
amikacin	3.1	96.3	2.5	97.2	2.3	97.7	↓	0.000
gentamicin	43.5	54.0	40.9	58.1	34.9	64.4	↓	0.000
ciprofloxacin	64.5	24.4	66.3	25.1	60.5	28.9	↓	0.000
levofloxacin	54.4	36.0	51.1	38.4	50.9	38.9	↓	0.000
trimethoprim/sulfamethoxazole	60.3	39.7	57.7	42.3	54.0	46.0	↓	0.000
fosfomycin	0.4	95.1	2.2	95.4	3.1	95.7	↑	0.000
polymyxin B	0.5	99.5	3.1	96.9	0.7	99.3	↓	0.000
tigecycline	0	100	0	100	0	100	–	NA
*K. pneumoniae*								
MDR	42.2	–	40.7	–	39.9	–	↑	0.000
ESBL-*K. pneumoniae*	28.7	–	27.8	–	27.6	–	↓	0.001
CR-*K. pneumoniae*	22.3	–	22.4	–	19.6	–	↑	0.000
amoxicillin/clavulanic acid	31.8	58.8	37.0	55.0	40.9	46.5	↑	0.000
piperacillin/tazobactam	25.8	70.6	17.5	70.2	26.2	65.2	↑	0.000
cefoperazone/sulbactam^a^	27.5	67.6	27.3	66.4	26.3	69.5	↑	0.000
ceftazidime/avibactam	–	–	–	–	1.6	98.4	–	NA
cefazolin	48.5	47.6	44.7	52.1	42.4	54.3	–	0.723
cefuroxime	45.3	51.9	43.7	53.7	42.9	54.3	–	0.094
ceftazidime	34.8	62	32.9	62.9	32.5	64.9	↑	0.001
ceftriaxone	44.6	54.9	41.3	57.7	39.6	59.8	–	0.309
cefepime	33.4	58.4	32.0	61.9	25.1	66.0	–	0.03
cefoxitin	29.9	66.4	30.1	66.9	27.7	67.7	↑	0.004
moxalactam	21.1	76.4	20.4	76.4	18.9	79.5	↑	0.000
aztreonam	36.7	60.0	36.0	61.8	35.3	62.7	↑	0.000
ertapenem	–	–	22.2	77.1	19.6	79.5	–	0.115
imipenem	21.1	77.4	21.4	77.6	19.4	80.1	↑	0.000
meropenem	20.3	78.9	21.4	78.3	19.5	80.4	↑	0.000
amikacin	16.7	83.1	14.6	85.2	14.7	85.1	↑	0.000
gentamicin	31.6	67.1	27.7	71.7	26.8	72.6	–	0.036
ciprofloxacin	51.7	46.6	44.0	50.3	44.7	52.8	–	0.065
levofloxacin	39.5	51.0	34.9	53.9	35.7	55.5	↑	0.000
trimethoprim/sulfamethoxazole	44.6	55.4	36.3	63.7	37.0	63.0	–	0.147
fosfomycin	5.7	89.2	9.4	86.2	7.7	90.3	↑	0.000
polymyxin B	1.0	99.0	9.0	91.0	1.6	98.4	–	0.937
tigecycline	0	98.8	0.1	98.6	0.1	99.9	↓	0.001
*E. cloacae*								
MDR	16.5		12.0		22.6		–	0.142
piperacillin/tazobactam	4.4	75.8	16.5	75.9	14.7	79.6	–	0.176
cefoperazone/sulbactam^a^	4.4	79.1	10.8	82.9	10.6	81.9	–	0.868
ceftazidime/avibactam	–	–	–	–	6.0	94.0	–	NA
ceftazidime	33.0	62.6	27.2	70.3	32.5	64.9	–	0.229
ceftriaxone	44.0	56.0	32.9	66.5	38.5	60.4	–	0.039
cefepime	11.0	82.4	13.3	82.3	16.6	74.7	–	0.039
moxalactam	6.6	83.5	4.4	86.7	7.9	83.8	–	0.256
aztreonam	36.3	61.5	27.8	71.5	30.6	66.8	–	0.531
ertapenem	–	–	4.4	93.7	6.0	91.7	–	0.481
imipenem	6.6	93.4	4.4	92.4	5.3	94.3	–	0.437
meropenem	5.5	94.5	3.8	96.2	5.7	94.3	–	0.130
amikacin	2.2	96.7	0.6	99.4	2.3	97.4	–	0.262
gentamicin	14.3	79.1	11.4	87.3	18.1	78.9	–	0.156
ciprofloxacin	24.2	68.1	21.5	72.2	26.8	70.2	–	0.909
levofloxacin	17.6	75.8	17.7	77.2	15.5	73.2	–	0.125
trimethoprim/sulfamethoxazole	26.4	73.6	15.2	84.8	27.5	72.5	–	0.068
fosfomycin	0	96.7	2.5	94.3	1.5	94.0	–	0.084
polymyxin B	37.4	62.6	43.0	57.0	36.2	63.8	↑	0.000
tigecycline	0	98.9	0	96.2	0	99.6	↓	0.000
*Klebsiella* spp								
MDR	11.9		14.9		11.5		↓	0.000
Piperacillin/tazobactam	15.3	76.3	21.6	63.5	20.0	74.6	–	0.266
Cefoperazone/Sulbactam^a^	16.9	81.4	18.9	73.0	11.5	83.8	–	0.033
Ceftazidime/avibactam	–	–	–	–	4.6	95.4	–	NA
Cefazolin	41.9	38.7	40.6	37.5	30.5	42.4	↑	0.000
Cefuroxime	22.6	74.2	40.5	55.4	18.6	78.0	↓	0.000
Ceftazidime	22.0	72.9	37.8	62.2	24.6	73.1	–	0.445
Ceftriaxone	32.2	66.1	40.5	56.8	33.8	63.8	↓	0.000
Cefepime	10.2	88.1	20.3	77.0	9.2	85.4	↓	0.019
Cefoxitin	16.1	77.4	21.9	68.8	11.9	84.7	↓	0.000
Moxalactam	8.5	89.8	9.5	83.8	7.8	88.4	–	0.052
Aztreonam	22.0	74.6	31.1	66.2	25.4	73.1	↓	0.000
Ertapenem	–	–	10.8	82.4	5.4	93.1	↓	0.046
Imipenem	10.2	93.2	9.5	82.4	4.6	93.1	↑	0.004
Meropenem	5.1	96.6	10.8	87.8	3.8	96.2	↑	0.000
Amikacin	3.4	91.5	1.4	98.6	0.8	99.2	↓	0.000
Gentamicin	6.8	66.1	12.2	87.8	4.6	93.8	↑	0.000
Ciprofloxacin	28.8	76.3	25.7	68.9	17.7	81.5	↑	0.000
Levofloxacin	13.6	91.5	24.3	70.3	12.3	84.6	↑	0.000
Trimethoprim/Sulfamethoxazole	8.5	100	10.8	89.2	16.2	83.8	↓	0.000
Fosfomycin	0	84.7	5.4	89.2	3.1	95.4	↑	0.000
Polymyxin B	15.3	100	13.5	86.5	0.8	99.2	↑	0.000
Tigecycline	0	96.2	0	98.6	0	99.2	↓	0.000
*Salmonella* spp								
MDR	63.0	–	35.7	–	52.1	–	↑	0.042
Amoxicillin	74.1	25.9	53.6	41.7	65.8	34.2	↑	0.000
Amoxicillin/Clavulanic Acid	9.3	38.9	8.1	61.9	6.0	47.9	–	0.943
Piperacillin/Tazobactam	0	98.1	2.4	88.1	0.9	99.1	–	0.128
Cefoperazone/Sulbactam^a^	1.9	96.3	4.8	92.9	0	98.3	↓	0.000
Ceftazidime/Avibactam	–	–	–		0	100	–	NA
Ceftazidime	18.5	81.5	9.5	90.5	8.5	91.5	–	0.904
Ceftriaxone	22.2	77.8	10.7	89.3	8.5	91.5	–	0.833
Cefepime	13.0	77.8	4.8	92.9	5.1	94.0	↑	0.000
Moxalactam	1.9	87.0	2.4	96.4	0	99.1	–	0.079
Aztreonam	0	68.5	9.5	89.3	8.5	91.5	–	0.916
Ertapenem	–	–	1.2	98.8	0	100	–	0.156
Imipenem	0	98.1	1.2	98.8	0	100	–	0.059
Meropenem	1.0	87	1.2	98.8	0	100	↓	0.008
Amikacin	1.9	98.1	1.2	97.6	0.9	99.1	–	0.192
Gentamicin	7.4	87.0	4.8	95.2	10.3	89.7	–	0.236
Ciprofloxacin	22.2	22.2	19.0	31.0	12.0	35.0	–	0.193
Levofloxacin	3.7	61.1	8.3	23.8	15.4	28.2	↑	0.000
Trimethoprim/Sulfamethoxazole	20.4	79.6	14.3	85.7	15.4	84.6	–	0.091
Fosfomycin	0	100	1.2	98.8	0.9	98.3	↑	0.002
Polymyxin B	55.6	44.4	36.9	63.1	53.8	46.2	↑	0.000
Tigecycline	7.4	92.6	0	100	0	100	–	0.813
*Serratia* spp								
MDR	12.8	–	5.8	–	5.9	–	↓	0.016
Piperacillin/Tazobactam	10.3	85.1	5.8	90.7	3.6	91.7	↓	0.000
Cefoperazone/Sulbactam^a^	12.8	82.1	32.6	64.0	8.4	88.0	–	0.103
Ceftazidime/Avibactam	–	–	–	–	0	100	–	NA
Ceftazidime	10.3	88.1	1.2	96.5	15.9	84.1	↓	0.000
Ceftriaxone	33.3	74.6	37.2	62.8	19.3	80.7	↓	0.003
Cefepime	15.4	80.6	32.6	65.1	9.5	83.3	↓	0.043
Moxalactam	12.8	94.0	0	98.8	3.6	95.2	↓	0.001
Aztreonam	20.5	76.1	11.6	60.5	11.9	83.3	↓	0.000
Ertapenem	–	–	2.3	96.5	8.4	91.6	↑	0.015
Imipenem	17.9	94.0	2.3	88.4	8.3	90.5	↓	0.000
Meropenem	10.3	94.0	2.3	97.7	8.3	91.7	↓	0.000
Amikacin	5.1	97.0	0	100	1.2	98.8	↓	0.018
Gentamicin	28.2	79.1	29.1	70.9	7.1	92.9	↓	0.004
Ciprofloxacin	36.8	47.8	41.9	40.7	16.7	57.1	–	0.930
Levofloxacin	35.9	70.1	36.0	52.3	13.1	82.1	–	0.890
Trimethoprim/Sulfamethoxazole	10.3	88.1	1.2	98.8	0	100	↓	0.000
Fosfomycin	0	100	2.3	97.7	0	100	↑	0.020
Tigecycline	5.1	98.5	0	98.8	0	100	↓	0.000

MDR, multidrug-resistance; ESBL-*E. coli*, extended-spectrum β-lactamase-producing *E. coli*; ESBL-*K. pneumoniae*, extended-spectrum β-lactamase-producing *K. pneumoniae*; CR-*E. coli*, carbapenem-resistant *E. coli*; CR-*K. pneumoniae*, carbapenem-resistant *K. pneumoniae*; NA, no account. ↑, resistance trend with increase. ↓, resistance trend with decrease. -, no significant change

^a^Criteria as published by the CLSI [8] for cefoperazone also applied to cefoperazone-sulbactam

The prevalence of ESBL-positive *E*. *coli* (ESBL-*E. coli*) and ESBL-positive *K. pneumonia* (ESBL-*K. pneumonia*) declined over the period of the study (both *p* < 0.05) (Table 2, Fig. 2). In general, higher resistance rates of pathogens were more common in tertiary hospitals compared with non-tertiary hospitals (Additional file 1: Table S2). However, the prevalence of ESBL-*E. coli* and ESBL-*K. pneumonia* was higher in developing regions compared with developed regions (58.2% and 36.4% vs 48.8% and 29.6%, respectively; both *p* < 0.05) (Additional file 1: Table S4).

Other Enterobacteriaceae, including *E. cloacae*, *Klebsiella* species (excluding *K. pneumoniae*), *Proteus* species, *Serratia* species, *Salmonella* species, and *Citrobacter* species showed lower resistance to ceftazidime/avibactam, carbapenem, and amikacin (Table 2, Additional file 1: Table S5).

#### Resistance in glucose non-fermenting Gram-negative bacteria

MDR rates in *A. baumannii* and *P. aeruginosa* were 70.3% and 21.1%, respectively, which did not increase significantly in 6 years (*p* = 0.841and *p* = 0.488, respectively) (Table 3). However, the PDR rate in *A. baumannii* was more than 50% (52.5%, 558/1062), wherein 6.7% of the strains were resistant to polymyxin B and 4.3% to tigecycline (Table 1). CR-*A. baumannii* (CR-AB) fluctuated between a prevalence of 55.7% and 71.2% during the surveillance time (Table 1, Fig. 2). The resistance rates in *A. baumannii* and *P. aeruginosa* were higher in tertiary hospitals than non-tertiary hospitals (Additional file 1: Table S2). It noted that the prevalence of CR*-A. baumannii* and CR-*P. aeruginosa* were higher in tertiary hospitals settings (71.3% and 25.5%, respectively, vs 52.0% and 12.6%, respectively; both *p* < 0.05) (Additional file 1: Table S4). However, the prevalence of CR*-A. baumannii* was higher in developing areas than developed areas (72.9% vs 56.7%, respectively; *p* < 0.05) (Additional file 1: Table S4).

**Table 3:** The susceptibility and resistance trends of non-fermentative pathogens to antimicrobial agents

	All strains (204–2019)					2014		2015		2016	
	Number	MIC_50_	MIC_90_	%R	%S	%R	%S	%R	%S	%R	%S
*A. baumannii*											
MDR	1062	–	–	70.3	–	63.6	–	67.6	–	74.3	–
piperacillin/tazobactam	1062	128	128	55.9	29.7	43.2	53.4	63.7	34.3	45.9	27.6
cefoperazone/sulbactam^a^	1062	64	128	65.6	31.0	58.0	39.8	62.7	34.3	72.0	26.1
ceftazidime	1062	64	128	66.4	32.6	61.4	37.5	64.7	32.4	72.8	26.5
cefepime	1062	64	64	65.3	31.3	56.8	40.9	64.7	33.3	63.8	28.8
imipenem	1062	32	64	63.6	35.9	52.3	46.6	60.8	39.2	71.2	28.8
meropenem	1062	32	64	63.6	35.5	54.5	45.5	59.8	38.2	69.6	28.8
amikacin	1062	64	128	51.5	46.4	46.6	51.1	50.0	50.0	53.3	44.7
gentamicin	1062	32	128	60.4	35.6	56.8	38.6	61.8	38.2	61.9	35.8
ciprofloxacin	1062	32	64	65.2	33.9	56.8	43.2	63.7	36.3	69.6	30.0
levofloxacin	1062	8	32	58.6	35.1	54.5	40.9	50.0	37.3	63.4	30.4
trimethoprim/sulfamethoxazole	1062	4	8	53.8	46.2	46.6	53.4	59.8	40.2	61.1	38.9
polymyxin B	1062	1	2	6.7	93.3	1.1	98.9	2.0	98	12.8	87.2
tigecycline	1062	1	4	4.3	86.5	4.5	92	4.9	90.2	6.6	81.7
*P. aeruginosa*											
MDR	–	–	–	21.1	–	22	–	22.8	–	21.2	–
piperacillin/tazobactam	1044	2	128	10.4	84.5	8.5	81.7	5.9	89.1	9.3	84.5
cefoperazone/sulbactam^a^	1044	8	64	12.4	79.9	13.4	73.2	6.9	84.2	11.9	78.8
ceftazidime/avibactam	362	4	8	2.8	97.2	–		–		–	
ceftazidime	1044	4	32	10.2	85.7	9.8	85.4	6.9	89.1	7.3	88.1
cefepime	1044	2	16	7.8	88.7	11.0	85.4	3.0	94.1	5.2	90.7
aztreonam	1044	4	32	14.4	76.9	12.2	78.0	12.9	78.2	10.4	79.8
imipenem	1044	2	32	18.8	79.9	13.5	70.7	12.9	57.4	18.7	80.3
meropenem	1044	0.25	16	13.5	83.2	6.1	92.7	9.9	86.1	10.9	86.5
amikacin	1044	2	8	2.2	97.2	3.7	96.3	3.0	95.0	3.1	96.4
gentamicin	1044	2	8	5.7	89.2	8.5	89.0	8.9	89.1	4.7	91.2
ciprofloxacin	1044	0.25	8	18.2	75.9	12.2	82.9	9.9	86.1	13.5	84.5
levofloxacin	1044	0.5	8	17.2	72.0	18.3	76.8	7.9	84.2	11.9	79.3
polymyxin B	1044	1	2	0	96.5	0	100	0	93.4	0	100

	2017		2018		2019		Trend	*p*
	%R	%S	%R	%S	%R	%S		
*A. baumannii*								
MDR	70.4	–	72.6	–	68.2	–	–	0.841
piperacillin/tazobactam	52.2	35.7	73.6	26.0	54.8	22.9	↑	0.005
cefoperazone/sulbactam^a^	66.1	33.9	68.8	28.4	61.0	32.2	–	0.664
ceftazidime	65.2	33.9	67.3	32.7	62.7	36.0	–	0.438
cefepime	63.5	33.0	69.7	28.8	67.1	30.8	–	0.062
imipenem	63.5	34.8	66.8	33.2	58.9	40.1	–	0.858
meropenem	65.2	34.8	66.8	32.2	59.2	40.1	–	0.920
amikacin	60.0	29.6	52.9	46.2	47.6	52.1	–	0.749
gentamicin	71.3	19.1	62.0	32.7	54.1	42.1	–	0.242
ciprofloxacin	67.8	29.6	69.7	28.8	59.9	39.0	–	0.763
levofloxacin	53.9	33.9	61.1	29.8	58.6	41.1	–	0.428
trimethoprim/sulfamethoxazole	50.4	49.6	56.7	43.3	46.6	53.4	–	0.070
polymyxin B	18.3	81.7	4.8	95.2	1.4	98.6	–	0.046
tigecycline	2.6	93	5.3	88.5	2.1	83.9	–	0.081
*P. aeruginosa*								
MDR	18	–	22.1	–	21.8	–	–	0.488
piperacillin/tazobactam	8.2	84.4	11.4	82.6	13.0	84.8	–	0.028
cefoperazone/sulbactam^a^	10.7	81.1	14.1	82.1	13.5	79.3	–	0.236
ceftazidime/avibactam	–		–		2.8	97.2	–	NA
ceftazidime	9.0	86.1	12.5	85.3	11.9	83.7	–	0.066
cefepime	9.0	88.5	8.7	88.0	8.8	87.3	–	0.258
aztreonam	14.8	78.7	15.8	73.9	16.6	75.7	–	0.067
imipenem	16.4	82.8	22.8	37.5	19.1	79.8	–	0.345
meropenem	12.3	86.1	15.8	78.8	16.9	79.8	↑	0.001
amikacin	4.9	94.3	1.6	97.8	0.6	99.2	–	0.012
gentamicin	8.2	86.9	6.5	90.8	3.3	88.1	–	0.029
ciprofloxacin	9.8	89.3	20.1	69.0	26.2	65.7	↑	0.000
levofloxacin	11.5	82.0	21.7	57.6	22.1	67.7	↑	0.001
polymyxin B	0	100	0	93.7	0	99.7	–	NA

MDR, multidrug-resistance; NA, no account. ↑, resistance trend with increase. ↓, resistance trend with decrease. –, no significant change

^a^Criteria as published by the CLSI [8] for cefoperazone also applied to cefoperazone-sulbactam

Cefoperazone/sulbactam and levofloxacin were the most effective agents against *Stenotrophomonas maltophilia* (with 95.0% and 89.2% susceptibility, respectively) (Additional file 1: Table S5). Overall, 18.3% of *S. maltophilia* were resistant to trimethoprim/sulfamethoxazole. The prevalence of ceftazidime-resistant *Burkholderia* species was 14.3%, and 24.4% of the strains were resistant to trimethoprim/sulfamethoxazole (Additional file 1: Table S5).

## Discussion

During the surveillance period from 2014 to 2019, *E. coli* and *K. pneumoniae* were the main BSI pathogens and the proportion of MRSA and ESBL-*E. coli* declined, while the frequency of CR-*K. pneumoniae* continuously increased. We also determined that the prevalence of antimicrobial resistant pathogens, especially Gram-negative organisms (GNO), varied by hospital types and the levels of local economic development. The declining trend could be attributed to the results of a special national campaign of antimicrobial stewardship initiated in 2012 [14]. As the first national, infection-defined, surveillance program in China, the BRICS has provided more precise data to help clinicians to improve antimicrobial therapy and contain the spread of AMR.

The frequency of blood cultures is recommended for 100 to 200 blood cultures sets per 1,000 patient days [15]. The 2018 annual report of the European Antimicrobial Resistance Surveillance Network (EARS-Net) varied substantially between countries, specifying only 5.3 blood cultures per 1,000 patient days in Lithuania, compared to 206.9 in Portugal [16]. However, 100 to 200 blood cultures sets per 1,000 patient days are far from routine use in China, as the China Antimicrobial Resistance Surveillance System shows that blood culture samples account for less than 10% of total microbiological samples [17]. In China, clinical microbiology is only a branch of laboratory medicine, which is different from established clinical microbiology and infectious disease departments in other countries. As a bloodstream-infection-definition surveillance, BRICS carried out AMR studies to raise attention to blood culture and promote antimicrobial stewardship programs by providing precisive surveillance data.

The surveillance revealed that GNO constituted a major proportion of all BSI causes, which increased during the monitoring period. Furthermore, the proportion of *E. coli* increased rapidly, which is consistent with previous reports [18]. The SENTRY Antimicrobial Surveillance Program also revealed that detection of GNO had increased and the proportion of GNO, such as *E. coli* and *K. pneumoniae*, also increased [19]. The emergence of MDR GNO, such as carbapenem-resistant strains, has increased rapidly [20], which makes treatment options extremely limited in clinical practice [21] as antibiotic development is lagging behind resistance for GNO. The spillover of the resistant bacteria, which is a consequence of the fact that antibiotic-resistant bacteria can be transmitted from person to person, could be the reasons for the increasing proportion of GNO [22].

During this surveillance, it is interesting to note that MRSA decreased over time, which is in line with other surveillance results [23]. Lawes et al. suggested that antibiotic stewardship and infection control strategies might have played an important part in the reduced prevalence of MRSA [24]. However, these strategies do not fully explain why pathogens other than MRSA are becoming more prevalent rather than declining over the same period. Although specific factors responsible for changes in the rates of MRSA infection remain uncertain, molecular epidemiology might provide a better understanding of MRSA population dynamics. The phenomenon of clonal replacement, whereby clones that were once widely disseminated during a certain period may become less dominant and are replaced by other epidemic clones, has been observed worldwide [25, 26]. In a Portuguese tertiary hospital, the Brazilian (ST239-IIIA) clone was replaced by the arrival of epidemic EMRSA-15 (ST22-IV) [27]. In China, the structural change in the population of MRSA was also observed when ST5-t437 replaced ST239-t030 as the predominant genotype [28].

Although the prevalence of ESBL-*E. coli* declined during the surveillance period, it was still at a high level, with a prevalence of more than 50%. This scenario has led to subsequent increased use of carbapenems, which was associated with the emergence and spread of carbapenem-resistant bacteria, especially among *K. pneumoniae* [29, 30]. This phenomenon was reflected in our monitoring data in which the CR-*K. pneumoniae* prevalence increased quickly. Other studies in China also indicated that CR-*K. pneumoniae* have reached higher epidemic levels in China [31, 32]. Furthermore, evidence of a particularly higher percentage of ST11 CR-*K. pneumoniae* [33] indicated there was clonal spread of CR-*K. pneumoniae* in hospitals. Our previous study also confirmed that ST11 KPC-2-producing *K. pneumoniae* was the common sequence types (STs) among carbapenemase-producing Enterobacteriaceae (CPE) [34]. However, sequencing was not carried out on all isolated carbapenem-resistant Enterobacteriaceae, which needs to be further studied. Accordingly, it is necessary to implement an antimicrobial stewardship program and effective infection control to contain and mitigate the risks of nosocomial transmission and outbreaks in hospitals, such as hand-hygiene education programs, contact precautions, and use of alert codes to promptly identify patients with CR-*K. pneumoniae* infections [35].

The CR-*A. baumannii* remained around 60% during the surveillance period, which poses a great challenge to patient treatment, and the prevalence was similar with other surveillance programs [36]. CR-*A. baumannii* is an emerging concern due to the associated high mortality rates [32]. The optimal antibiotic choice for CR-*A. baumannii* bacteremia is controversial. Some studies have suggested the use of polymyxin as the backbone of treatment [37, 38], Only colistin or tigecycline still presented higher susceptibility rates in this surveillance; however, the clinical efficacy of the two agents still needs to be confirmed. Hospitals should consider antimicrobial stewardship and infection control when tackling CR-*A. baumannii*. Indeed, Latibeaudiere et al. demonstrated that previous CR-*A. baumannii* colonization increased the risk of developing a CR-*A. baumannii* infection by eightfold [39]. Targeted antibacterial therapy against infection; rather than colonization and promoting hand hygiene, environmental cleaning, and contact precautions; could be valuable strategies in the control of CR-*A. baumannii* [40].

It is conceivable that resistance rates are higher in tertiary hospitals where patients with more critical conditions are admitted; larger scale operations, more frequent organ transplantations, and longer hospitalizations increase a patient’s risk of infection, which leads to more antibiotic use and easier induction of AMR [41]. Nonetheless, the higher AMR observed in developing regions may result from a poorer healthcare infrastructure, weaker infection control implementation, and less common antimicrobial stewardship activity. All hospitals should take individual actions to contain the spread of AMR.

There are some limitations to note in this study. First, participating hospitals covered 18 of the 31 provinces in mainland China. However, it has not yet covered all provinces and the participating hospitals are only a small proportion of the total hospitals in China. Therefore, it must be noted that by increasing the number of participants, the surveillance data might become more precise. Second, the failure to distinguish the pathogens isolated from community-acquired or hospital-acquired BSI was a major limitation. In China, blood culture is mainly requested for patients with symptoms of infection during hospitalization, which means most BSI is nosocomial BSI. Third, some relevant denominators, like patient-days, blood culture rates and patient characteristics, were not recorded for all patients. Last, this surveillance is not a population-based surveillance, and the incidence of BSI is not available.

## Conclusions

In this report of the first national BSI surveillance program in China, *E. coli* and *K. pneumoniae* were the main BSI pathogens. The proportion of MRSA and ESBL-*E. coli* declined, while the frequency of CR- *K. pneumoniae* continuously increased. The prevalence of antimicrobial-resistant pathogens, especially ESBL-*E. coli*, CR-*E. coli*, ESBL-*K. pneumonia*, CR-*K. pneumonia* and CR-*A. baumannii*, varied by hospital types and the level of local economic development.

## Supplementary Information

**Additional file 1. Supplemental Table 1**. Rank order of pathogens causing bloodstream infection nationwide submitted to BRICS during 2014-2019, by hospital type and region economic development.** Supplemental Table 2**. The resistance of major pathogensto antimicrobial agentsby hospital level and region economic development.** Supplemental Table 3**. The susceptibility and resistanceof MRSA and MRCNSto antimicrobial agents.** Supplemental Table 4**. TheMDR prevalence ofmajor pathogens to antimicrobial agents by hospital level and region economic development.** Supplement Table 5**. The susceptibility and resistanceof other pathogensto antimicrobial agents.** Supplemental Table 6**. The susceptibility and resistanceof ESBL+, ESBL-and CR-*E. colito* antimicrobial agent.** Supplemental Table 7**. The susceptibility and resistanceof ESBL+, ESBL-and CR-K.* pneumoniaeto* antimicrobial agents.

## Data Availability

Not applicable.
