# Supplementary material for: Blood bacterial resistant investigation collaborative system (BRICS) report: a national surveillance in China from 2014 to 2019
Source: Antimicrob Resist Infect Control. 2022 Jan 24;11:17. doi: 10.1186/s13756-022-01055-5 (PMC8785473; doi:10.1186/s13756-022-01055-5)
Supplement: Supplementary file 1 — Additional file 1. Supplemental Table 1. Rank order of pathogens causing bloodstream infection nationwide submitted to BRICS during 2014-2019, by hospital type and region economic development. Supplemental Table 2. The resistance of major pathogensto antimicrobial agentsby hospital level and region economic development. Supplemental Table 3. The susceptibility and resistanceof MRSA and MRCNSto antimicrobial agents. Supplemental Table 4. TheMDR prevalence ofmajor pathogens to antimicrobial agents by hospital level and region economic development. Supplement Table 5. The susceptibility and resistanceof other pathogensto antimicrobial agents. Supplemental Table 6. The susceptibility and resistanceof ESBL+, ESBL-and CR-E. colito antimicrobial agent. Supplemental Table 7. The susceptibility and resistanceof ESBL+, ESBL-and CR-K. pneumoniaeto antimicrobial agents. [file 13756_2022_1055_MOESM1_ESM.docx]

Supplemental Table 1 Rank order of pathogens causing bloodstream infection nationwide submitted to BRICS during 2014-2019, by hospital type and region economic development

| Rank | Pathogen (%) for hospital type | | Pathogen (%) for region development | |
| --- | --- | --- | --- | --- |
|  | Tertiary hospitals | Secondary hospitals | Developed regions | Developing regions |
| 1 | *E. coli* (32.5) | *E. coli* (38.8) | *E. coli* (32.5) | *E. coli* (36.9) |
| 2 | *K. pneumoniae* (16.1) | *K. pneumoniae* (15.2) | *K. pneumoniae* (16.8) | *K. pneumoniae* (14.4) |
| 3 | coagulase negative *Staphylococcus* (10.1) | *S. aureus* (11.5) | *S. aureus* (10.4) | coagulase negative *Staphylococcus* (12.1) |
| 4 | *S. aureus* (9.0) | coagulase negative *Staphylococcus* (9.4) | coagulase negative *Staphylococcus* (7.7) | *S. aureus* (9.9) |
| 5 | *A. baumannii (4.7)* | *P. aeruginosa* (3.2) | *A. baumannii (3.9)* | *A. baumannii (3.8)* |
| 6 | *P. aeruginosa* (4.3) | *A. baumannii (2.9)* | *P. aeruginosa* (3.9) | *P. aeruginosa* (3.6) |
| 7 | *E. faecium* (4.1) | *E. faecium* (2.6) | *E. faecium* (3.6) | *E. faecium* (3.2) |
| 8 | *E. cloacae* (3.2) | *E. cloacae* (2.4) | *E. cloacae* (2.7) | *E. cloacae* (2.9) |
| 9 | *E. faecalis* (2.4) | *E. faecalis* (2.1) | *E. faecalis* (2.5) | *E. faecalis* (2.0) |
| 10 | 𝝰-hemolytic S*treptococci* (1.7) | 𝝱-hemolytic S*treptococci* (1.5) | 𝝱-hemolytic S*treptococci* (1.7) | *Klebsiella sp.* (1.5) |
| 11 | Others (2.8) | Others (2.4) | Others (3.4) | Others (2.4) |

Supplemental Table 2 The resistance of major pathogens to antimicrobial agents by hospital level and region economic development

| Antibiotics | Total |  | Tertiary hospitals |  | Non-tertiary hospitals |  | Developed Regions |  | Developing Regions |  |
| --- | --- | --- | --- | --- | --- | --- | --- | --- | --- | --- |
|  | Number | %R | Number | %R | Number | %R | Number | %R | Number | %R |
| *S. aureu* | | | | | | | | | | |
| MRSA | 2843 | 31.3 | 1285 | 31.0 | 1558 | 31.6 | 1541 | 33.9 | 1302 | 28.3 |
| penicillin G | 2667 | 87.9 | 1215 | 88.3 | 1452 | 87.5 | 1444 | 86.5 | 1223 | 89.5 |
| oxacillin | 2843 | 31.3 | 1285 | 31.0 | 1558 | 31.6 | 1541 | 33.9 | 1302 | 28.3 |
| amikacin | 2843 | 3.7 | 1285 | 3.3 | 1558 | 3.9 | 1541 | 3.4 | 1302 | 4.0 |
| gentamicin | 2360 | 15.3 | 1090 | 15.9 | 1270 | 14.9 | 1304 | 17.3 | 1056 | 13.0 |
| rifampicin | 2843 | 3.0 | 1285 | 3.2 | 1558 | 2.8 | 1541 | 1.8 | 1302 | 4.5 |
| ciprofloxacin | 2360 | 21.3 | 1090 | 23.1 | 1270 | 19.7 | 1304 | 23.3 | 1056 | 18.8 |
| levofloxacin | 2843 | 17.9 | 1285 | 18.5 | 1558 | 17.4 | 1541 | 20.3 | 1302 | 15.1 |
| moxifloxacin | 2843 | 17.6 | 1285 | 18.0 | 1558 | 17.3 | 1541 | 19.7 | 1302 | 15.1 |
| trimethoprim/sulfamethoxazole | 2843 | 2.8 | 1285 | 3.3 | 1558 | 2.5 | 1541 | 2.5 | 1302 | 3.2 |
| clindamycin | 2843 | 37.0 | 1285 | 38.2 | 1558 | 35.9 | 1541 | 39.0 | 1302 | 34.6 |
| erythromycin | 2843 | 60.1 | 1285 | 60.5 | 1558 | 59.8 | 1541 | 60.4 | 1302 | 59.8 |
| daptomycin | 2843 | 0.0 | 1285 | 0 | 1558 | 0.0 | 1541 | 0 | 1302 | 0 |
| linezolid | 2843 | 0.3 | 1285 | 0.2 | 1558 | 0.4 | 1541 | 0.4 | 1302 | 0.2 |
| vancomycin | 2843 | 0.0 | 1285 | 0 | 1558 | 0 | 1541 | 0 | 1302 | 0 |
| teicoplanin | 2360 | 0.0 | 1090 | 0 | 1270 | 0 | 1304 | 0 | 1056 | 0 |
| tetracycline | 2843 | 19.4 | 1285 | 17.5 | 1558 | 21.0 | 1541 | 20.9 | 1302 | 17.7 |
| tigecycline | 2843 | 1.2 | 1285 | 0.8 | 1558 | 1.5 | 1541 | 1.1 | 1302 | 1.3 |
| coagulase-negative *Staphylococci* | | | | | | | | | | |
| MRCNS | - | 74.3 | - | 75.9 | - | 72.4 | - | 70.7 | - | 76.8 |
| penicillin G | 1832 | 87.8 | 989 | 88.1 | 851 | 87.3 | 836 | 85.6 | 1004 | 89.4 |
| oxacillin | 2713 | 74.3 | 1451 | 75.9 | 1269 | 72.4 | 1129 | 70.7 | 1591 | 76.8 |
| amikacin | 2713 | 4.9 | 1451 | 5.0 | 1269 | 4.6 | 1129 | 5.1 | 1591 | 4.7 |
| gentamicin | 1832 | 37.1 | 989 | 36.2 | 850 | 38.0 | 835 | 35.8 | 1004 | 38.0 |
| rifampicin | 2713 | 6.9 | 1451 | 7.0 | 1269 | 6.7 | 1129 | 5.8 | 1591 | 7.7 |
| ciprofloxacin | 1832 | 45.4 | 989 | 46.4 | 850 | 44.0 | 835 | 45.6 | 1004 | 45.0 |
| levofloxacin | 2713 | 45.6 | 1451 | 46.2 | 1270 | 44.9 | 1130 | 46.1 | 1591 | 45.3 |
| moxifloxacin | 2713 | 40.0 | 1451 | 40.7 | 1270 | 39.2 | 1130 | 40.8 | 1591 | 39.4 |
| trimethoprim/sulfamethoxazole | 2713 | 30.5 | 1451 | 30.7 | 1270 | 30.1 | 1130 | 27.3 | 1591 | 32.7 |
| clindamycin | 2713 | 36.7 | 1451 | 38.4 | 1270 | 34.6 | 1130 | 37.0 | 1591 | 36.4 |
| erythromycin | 2713 | 78.5 | 1451 | 77.5 | 1270 | 79.4 | 1130 | 74.3 | 1591 | 81.3 |
| daptomycin | 2713 | 0.0 | 1451 | 0 | 1270 | 0 | 1130 | 0 | 1591 | 0 |
| linezolid | 2713 | 1.3 | 1451 | 1.4 | 1270 | 1.1 | 1130 | 1.9 | 1591 | 0.9 |
| vancomycin | 2713 | 0.0 | 1451 | 0 | 1270 | 0.0 | 1130 | 0 | 1591 | 0 |
| teicoplanin | 1832 | 0.0 | 989 | 0 | 851 | 0.0 | 836 | 0 | 1004 | 0 |
| tetracycline | 2713 | 24.2 | 1451 | 21.6 | 1270 | 27.2 | 1130 | 19.9 | 1591 | 27.2 |
| tigecycline | 2713 | 6.5 | 1451 | 5.7 | 1270 | 7.5 | 1130 | 6.4 | 1591 | 6.6 |
| *E. faecium* | | | | | | | | | | |
| penicillin G | 950 | 88.8 | 592 | 89.9 | 358 | 87.2 | 524 | 88.5 | 426 | 89.2 |
| ampicillin | 950 | 86.8 | 592 | 87.5 | 358 | 85.8 | 524 | 86.8 | 426 | 86.9 |
| rifampicin | 815 | 82.2 | 523 | 82.2 | 292 | 82.2 | 478 | 87.2 | 337 | 75.1 |
| ciprofloxacin | 815 | 91.9 | 523 | 92.2 | 292 | 91.4 | 478 | 92.9 | 337 | 90.5 |
| levofloxacin | 950 | 86.3 | 592 | 87.7 | 358 | 84.1 | 524 | 86.3 | 426 | 86.4 |
| erythromycin | 950 | 87.9 | 592 | 89.0 | 358 | 86.0 | 524 | 88.2 | 426 | 87.6 |
| daptomycin | 950 | 0.1 | 592 | 0 | 358 | 0.3 | 524 | 0 | 426 | 0.2 |
| linezolid | 950 | 0.5 | 592 | 0.2 | 358 | 1.1 | 524 | 0.2 | 426 | 0.9 |
| vancomycin | 949 | 0.5 | 592 | 0.5 | 357 | 0.6 | 524 | 0.2 | 425 | 0.9 |
| teicoplanin | 815 | 0.2 | 523 | 0.4 | 292 | 0.0 | 478 | 0 | 337 | 0.6 |
| tigecycline | 950 | 0.3 | 592 | 0.3 | 358 | 0.3 | 524 | 0.2 | 426 | 0.5 |
| *E. faecalis* | | | | | | | | | | |
| penicillin G | 636 | 9.0 | 350 | 9.7 | 286 | 8.0 | 370 | 7.8 | 266 | 10.5 |
| ampicillin | 636 | 7.4 | 350 | 6.3 | 286 | 8.7 | 370 | 5.1 | 266 | 10.5 |
| rifampicin | 549 | 50.1 | 307 | 47.9 | 242 | 52.9 | 327 | 48.9 | 222 | 51.8 |
| ciprofloxacin | 549 | 25.7 | 307 | 28.3 | 242 | 22.3 | 327 | 26.6 | 222 | 24.3 |
| levofloxacin | 636 | 26.1 | 350 | 27.4 | 286 | 24.5 | 370 | 25.7 | 266 | 26.7 |
| erythromycin | 636 | 58.3 | 350 | 61.1 | 286 | 54.9 | 370 | 54.3 | 266 | 63.9 |
| daptomycin | 636 | 0.3 | 350 | 0.3 | 286 | 0.3 | 370 | 0.5 | 266 | 0 |
| linezolid | 636 | 1.3 | 350 | 1.1 | 286 | 1.4 | 370 | 0.8 | 266 | 1.9 |
| vancomycin | 636 | 0.0 | 350 | 0 | 286 | 0 | 370 | 0.0 | 266 | 0 |
| teicoplanin | 549 | 0.2 | 307 | 0 | 242 | 0.4 | 327 | 0.0 | 222 | 0.5 |
| tigecycline | 636 | 0.6 | 350 | 0.9 | 286 | 0.3 | 370 | 0.8 | 266 | 0.4 |
| α-hemolytic Streptococci | | | | | | | | | | |
| penicillin G | 352 | 1.4 | 245 | 1.6 | 107 | 0.9 | 227 | 1.8 | 125 | 0.8 |
| ceftriaxone | 351 | 6.6 | 245 | 8.6 | 106 | 1.9 | 227 | 7.0 | 124 | 5.6 |
| cefepime | 290 | 7.2 | 208 | 9.6 | 82 | 1.2 | 211 | 8.5 | 79 | 3.8 |
| meropenem | 290 | 0.0 | 208 | 0 | 82 | 0 | 211 | 0 | 79 | 0 |
| levofloxacin | 352 | 11.9 | 245 | 12.2 | 107 | 11.2 | 227 | 13.2 | 125 | 9.6 |
| clindamycin | 352 | 58.0 | 245 | 57.6 | 107 | 58.9 | 227 | 55.9 | 125 | 61.6 |
| erythromycin | 352 | 69.3 | 245 | 68.2 | 107 | 72.0 | 227 | 68.7 | 125 | 70.4 |
| daptomycin | 352 | 0.0 | 245 | 0 | 107 | 0 | 227 | 0 | 125 | 0 |
| linezolid | 352 | 0.0 | 245 | 0 | 107 | 0 | 227 | 0 | 125 | 0 |
| vancomycin | 352 | 0.0 | 245 | 0 | 107 | 0 | 227 | 0 | 125 | 0 |
| teicoplanin | 146 | 0.0 | 101 | 0 | 45 | 0 | 100 | 0 | 46 | 0 |
| tetracycline | 352 | 41.2 | 245 | 38.4 | 107 | 47.7 | 227 | 42.7 | 125 | 38.4 |
| tigecycline | 206 | 1.5 | 137 | 2.2 | 69 | 0.0 | 116 | 0.0 | 90 | 3.3 |
| β-hemolytic Streptococci | | | | | | | | | | |
| penicillin G | 388 | 0.0 | 189 | 0 | 199 | 0 | 249 | 0 | 139 | 0 |
| ceftriaxone | 388 | 0.0 | 189 | 0 | 199 | 0 | 249 | 0 | 139 | 0 |
| cefepime | 332 | 0.0 | 159 | 0 | 173 | 0 | 224 | 0 | 108 | 0 |
| meropenem | 332 | 0.0 | 159 | 0 | 173 | 0 | 224 | 0 | 108 | 0 |
| levofloxacin | 388 | 25.0 | 189 | 27.0 | 199 | 23.1 | 249 | 25.7 | 139 | 23.7 |
| clindamycin | 388 | 73.2 | 189 | 73.5 | 199 | 72.9 | 249 | 71.5 | 139 | 76.3 |
| erythromycin | 388 | 80.7 | 189 | 78.8 | 199 | 82.4 | 249 | 79.5 | 139 | 82.7 |
| daptomycin | 388 | 0.0 | 189 | 0 | 199 | 0 | 249 | 0 | 139 | 0 |
| linezolid | 388 | 0.0 | 189 | 0 | 199 | 0 | 249 | 0 | 139 | 0 |
| vancomycin | 388 | 0.0 | 189 | 0 | 199 | 0 | 249 | 0 | 139 | 0 |
| teicoplanin | 182 | 0.0 | 95 | 0 | 87 | 0 | 121 | 0 | 61 | 0 |
| tetracycline | 238 | 46.2 | 125 | 51.2 | 113 | 40.7 | 146 | 48.6 | 92 | 42.4 |
| tigecycline | 388 | 3.9 | 189 | 2.6 | 199 | 5.0 | 249 | 4.4 | 139 | 2.9 |
| *E. coli* | | | | | | | | | | |
| CREC | 9944 | 1.5 | 4678 | 2.0 | 5266 | 1.0 | 5087 | 1.3 | 4857 | 1.6 |
| ESBL | 9944 | 53.4 | 4678 | 56.7 | 5266 | 50.4 | 5087 | 48.8 | 4857 | 58.2 |
| amoxicillin | 9944 | 74.8 | 4678 | 75.5 | 5266 | 74.2 | 5087 | 74.1 | 4857 | 75.5 |
| amoxicillin/clavulanic acid | 9944 | 39.3 | 4678 | 40.8 | 5266 | 38.0 | 5087 | 39.7 | 4857 | 38.9 |
| piperacillin/tazobactam | 9944 | 7.5 | 4678 | 8.4 | 5266 | 6.7 | 5087 | 7.1 | 4857 | 7.9 |
| cefoperazone-sulbactam^a^ | 9944 | 8.5 | 4678 | 10.1 | 5266 | 7.0 | 5087 | 7.6 | 4857 | 9.3 |
| ceftazidime/avibactam | 3683 | 1.0 | 1514 | 1.8 | 2169 | 0.4 | 2088 | 1.1 | 1595 | 0.9 |
| cefazolin | 9944 | 59.7 | 4678 | 63.8 | 5266 | 56.0 | 5087 | 55.5 | 4857 | 64.1 |
| cefuroxime | 9944 | 55.0 | 4678 | 58.6 | 5266 | 51.9 | 5087 | 51.0 | 4857 | 59.3 |
| ceftazidime | 9944 | 28.9 | 4678 | 32.4 | 5266 | 25.7 | 5087 | 25.0 | 4857 | 32.9 |
| ceftriaxone | 9944 | 55.8 | 4678 | 60.0 | 5266 | 52.1 | 5087 | 51.3 | 4857 | 60.6 |
| cefepime | 9944 | 21.0 | 4678 | 24.7 | 5266 | 17.8 | 5087 | 18.1 | 4857 | 24.1 |
| cefoxitin | 9944 | 13.6 | 4678 | 15.6 | 5266 | 11.8 | 5087 | 12.6 | 4857 | 14.6 |
| moxalactam | 9943 | 2.5 | 4678 | 3.3 | 5265 | 1.8 | 5087 | 2.3 | 4856 | 2.7 |
| aztreonam | 9944 | 36.2 | 4678 | 39.5 | 5266 | 33.3 | 5087 | 32.4 | 4857 | 40.3 |
| ertapenem | 5495 | 1.5 | 2408 | 2.2 | 3087 | 1.0 | 2951 | 1.5 | 2544 | 1.6 |
| imipenem | 9944 | 1.4 | 4678 | 1.9 | 5266 | 0.9 | 5087 | 1.3 | 4857 | 1.5 |
| meropenem | 9944 | 1.4 | 4678 | 1.8 | 5266 | 1.0 | 5087 | 1.3 | 4857 | 1.5 |
| amikacin | 9944 | 2.9 | 4678 | 3.4 | 5266 | 2.4 | 5087 | 2.7 | 4857 | 3.0 |
| gentamicin | 9944 | 39.5 | 4678 | 40.0 | 5266 | 39.1 | 5087 | 37.2 | 4857 | 41.9 |
| ciprofloxacin | 9944 | 64.3 | 4678 | 67.1 | 5266 | 61.9 | 5087 | 62.4 | 4857 | 66.4 |
| levofloxacin | 9944 | 53.3 | 4678 | 57.2 | 5266 | 49.9 | 5087 | 51.7 | 4857 | 55.1 |
| trimethoprim/sulfamethoxazole | 9944 | 57.1 | 4678 | 59.3 | 5266 | 55.1 | 5087 | 55.2 | 4857 | 59.1 |
| fosfomycin | 9944 | 1.7 | 4678 | 2.3 | 5266 | 1.2 | 5087 | 2.1 | 4857 | 1.3 |
| polymyxin B | 9944 | 2.2 | 4678 | 2.8 | 5266 | 1.7 | 5087 | 2.0 | 4857 | 2.4 |
| tigecycline | 9944 | 0.0 | 4678 | 0 | 5266 | 0.0 | 5087 | 0 | 4857 | 0 |
| *K. pneumonia* | | | | | | | | | | |
| CRKP | 4378 | 18.3 | 2310 | 25.6 | 2068 | 10.2 | 2481 | 20.3 | 1897 | 15.7 |
| ESBL | 4378 | 32.5 | 2310 | 36.6 | 2068 | 28.0 | 2481 | 29.6 | 1897 | 36.4 |
| amoxicillin/clavulanic acid | 3679 | 36.0 | 1937 | 44.7 | 1742 | 26.4 | 2116 | 37.5 | 1563 | 34.0 |
| piperacillin/tazobactam | 4378 | 22.0 | 2310 | 27.7 | 2068 | 15.6 | 2481 | 22.9 | 1897 | 20.9 |
| cefoperazone-sulbactam^a^ | 4378 | 24.9 | 2310 | 33.0 | 2068 | 15.8 | 2481 | 25.7 | 1897 | 23.8 |
| ceftazidime/avibactam | 1655 | 1.6 | 824 | 2.7 | 831 | 0.6 | 979 | 1.7 | 676 | 1.5 |
| cefazolin | 4378 | 43.5 | 2310 | 51.9 | 2068 | 34.2 | 2481 | 42.5 | 1897 | 44.9 |
| cefuroxime | 4378 | 42.7 | 2310 | 51.0 | 2068 | 33.5 | 2481 | 41.8 | 1897 | 43.9 |
| ceftazidime | 4378 | 31.6 | 2310 | 39.6 | 2068 | 22.6 | 2481 | 31.2 | 1897 | 32.0 |
| ceftriaxone | 4378 | 40.2 | 2310 | 48.8 | 2068 | 30.6 | 2481 | 38.9 | 1897 | 41.8 |
| cefepime | 4378 | 27.0 | 2310 | 36.1 | 2068 | 16.9 | 2481 | 27.0 | 1897 | 27.0 |
| cefoxitin | 4378 | 27.3 | 2310 | 35.3 | 2068 | 18.4 | 2481 | 27.9 | 1897 | 26.5 |
| moxalactam | 4378 | 17.4 | 2310 | 24.3 | 2068 | 9.7 | 2481 | 18.9 | 1897 | 15.4 |
| aztreonam | 4378 | 34.1 | 2310 | 41.9 | 2068 | 25.4 | 2481 | 33.3 | 1897 | 35.1 |
| ertapenem | 2518 | 20.5 | 1331 | 28.2 | 1187 | 12.0 | 1466 | 22.3 | 1052 | 18.1 |
| imipenem | 4378 | 17.5 | 2310 | 24.6 | 2068 | 9.6 | 2481 | 19.7 | 1897 | 14.7 |
| meropenem | 4378 | 17.4 | 2310 | 24.5 | 2068 | 9.5 | 2481 | 19.4 | 1897 | 14.8 |
| amikacin | 4378 | 13.7 | 2310 | 19.3 | 2068 | 7.4 | 2481 | 14.2 | 1897 | 13.0 |
| gentamicin | 4378 | 26.8 | 2310 | 33.1 | 2068 | 19.7 | 2481 | 26.2 | 1897 | 27.5 |
| ciprofloxacin | 4378 | 44.5 | 2310 | 50.6 | 2068 | 37.6 | 2481 | 44.1 | 1897 | 45.1 |
| levofloxacin | 4378 | 34.1 | 2310 | 41.6 | 2068 | 25.7 | 2481 | 35.3 | 1897 | 32.5 |
| trimethoprim/sulfamethoxazole | 4378 | 36.8 | 2310 | 41.0 | 2068 | 32.2 | 2481 | 36.9 | 1897 | 36.7 |
| fosfomycin | 4378 | 6.1 | 2310 | 8.4 | 2068 | 3.4 | 2481 | 7.3 | 1897 | 4.4 |
| polymyxin B | 4378 | 3.6 | 2310 | 3.8 | 2068 | 3.4 | 2481 | 2.7 | 1897 | 4.7 |
| tigecycline | 4378 | 0.2 | 2310 | 0.2 | 2068 | 0.2 | 2481 | 0.2 | 1897 | 0.3 |
| *E. cloacae* | | | | | | | | | | |
| piperacillin/tazobactam | 785 | 13.1 | 453 | 14.6 | 332 | 11.1 | 400 | 11.8 | 385 | 14.5 |
| cefoperazone-sulbactam^a^ | 785 | 9.8 | 453 | 11.3 | 332 | 7.8 | 400 | 8.2 | 385 | 11.4 |
| ceftazidime/avibactam | 265 | 6.0 | 154 | 9.1 | 111 | 1.8 | 148 | 6.1 | 117 | 6.0 |
| cefuroxime | 299 | 43.8 | 179 | 43.6 | 120 | 44.2 | 143 | 41.3 | 156 | 46.2 |
| ceftazidime | 785 | 29.6 | 453 | 32.5 | 332 | 25.6 | 400 | 25.0 | 385 | 34.3 |
| ceftriaxone | 785 | 39.9 | 453 | 42.2 | 332 | 36.7 | 400 | 33.2 | 385 | 46.8 |
| cefepime | 785 | 13.4 | 453 | 17.4 | 332 | 7.8 | 400 | 10.5 | 385 | 16.4 |
| moxalactam | 785 | 6.2 | 453 | 7.7 | 332 | 4.2 | 400 | 7.2 | 385 | 5.2 |
| aztreonam | 785 | 29.7 | 453 | 31.6 | 332 | 27.1 | 400 | 24.2 | 385 | 35.3 |
| ertapenem | 423 | 5.4 | 255 | 7.1 | 168 | 3.0 | 228 | 6.1 | 195 | 4.6 |
| imipenem | 785 | 4.8 | 453 | 5.7 | 332 | 3.6 | 400 | 4.8 | 385 | 4.9 |
| meropenem | 785 | 4.5 | 453 | 5.7 | 332 | 2.7 | 400 | 4.5 | 385 | 4.4 |
| amikacin | 785 | 2.3 | 453 | 2.6 | 332 | 1.8 | 400 | 2.2 | 385 | 2.3 |
| gentamicin | 785 | 14.5 | 453 | 15.9 | 332 | 12.7 | 400 | 9.5 | 385 | 19.7 |
| ciprofloxacin | 785 | 25.0 | 453 | 27.8 | 332 | 21.1 | 400 | 20.5 | 385 | 29.6 |
| levofloxacin | 785 | 17.7 | 453 | 18.8 | 332 | 16.3 | 400 | 15.8 | 385 | 19.7 |
| trimethoprim/sulfamethoxazole | 785 | 25.1 | 453 | 26.7 | 332 | 22.9 | 400 | 18.8 | 385 | 31.7 |
| fosfomycin | 785 | 1.1 | 453 | 0.9 | 332 | 1.5 | 400 | 1.5 | 385 | 0.8 |
| polymyxin B | 785 | 28.7 | 453 | 32.0 | 332 | 24.1 | 400 | 27.5 | 385 | 29.9 |
| tigecycline | 785 | 3.2 | 453 | 4.0 | 332 | 2.1 | 400 | 3.2 | 385 | 3.1 |
| *Klebsiella. spp.* | | | | | | | | | | |
| amoxicillin/clavulanic acid | 155 | 27.1 | 90 | 36.7 | 65 | 13.8 | 74 | 32.4 | 81 | 22.2 |
| piperacillin/tazobactam | 401 | 18.0 | 218 | 22.5 | 183 | 12.6 | 203 | 18.7 | 198 | 17.2 |
| ceftazidime/avibactam | 130 | 4.6 | 72 | 8.3 | 58 | 0.0 | 75 | 2.7 | 55 | 7.3 |
| cefazolin | 198 | 43.9 | 112 | 49.1 | 86 | 37.2 | 90 | 43.3 | 108 | 44.4 |
| cefuroxime | 266 | 38.0 | 141 | 42.6 | 125 | 32.8 | 128 | 33.6 | 138 | 42.0 |
| ceftazidime | 401 | 28.7 | 218 | 30.7 | 183 | 26.2 | 203 | 29.6 | 198 | 27.8 |
| ceftriaxone | 401 | 39.4 | 218 | 44.5 | 183 | 33.3 | 203 | 38.4 | 198 | 40.4 |
| cefepime | 401 | 15.0 | 218 | 18.3 | 183 | 10.9 | 203 | 13.8 | 198 | 16.2 |
| cefoxitin | 198 | 18.2 | 112 | 23.2 | 86 | 11.6 | 90 | 20.0 | 108 | 16.7 |
| moxalactam | 400 | 7.5 | 217 | 10.6 | 183 | 3.8 | 202 | 5.4 | 198 | 9.6 |
| aztreonam | 401 | 28.7 | 218 | 33.0 | 183 | 23.5 | 203 | 30.5 | 198 | 26.8 |
| ertapenem | 204 | 7.4 | 108 | 13.0 | 96 | 1.0 | 115 | 7.8 | 89 | 6.7 |
| imipenem | 401 | 6.2 | 218 | 9.6 | 183 | 2.2 | 203 | 5.9 | 198 | 6.6 |
| meropenem | 401 | 5.0 | 218 | 7.8 | 183 | 1.6 | 203 | 5.4 | 198 | 4.5 |
| amikacin | 401 | 2.5 | 218 | 2.3 | 183 | 2.7 | 203 | 1.0 | 198 | 4.0 |
| gentamicin | 401 | 12.0 | 218 | 11.5 | 183 | 12.6 | 203 | 9.9 | 198 | 14.1 |
| ciprofloxacin | 401 | 30.2 | 218 | 31.7 | 183 | 28.4 | 203 | 29.1 | 198 | 31.3 |
| levofloxacin | 401 | 21.2 | 218 | 23.4 | 183 | 18.6 | 203 | 18.2 | 198 | 24.2 |
| trimethoprim/sulfamethoxazole | 401 | 19.0 | 218 | 20.6 | 183 | 16.9 | 203 | 16.7 | 198 | 21.2 |
| fosfomycin | 401 | 2.5 | 218 | 2.8 | 183 | 2.2 | 203 | 3.4 | 198 | 1.5 |
| polymyxin B | 401 | 6.0 | 218 | 7.3 | 183 | 4.4 | 203 | 7.9 | 198 | 4.0 |
| tigecycline | 401 | 1.2 | 218 | 2.3 | 183 | 0.0 | 203 | 1.0 | 198 | 1.5 |
| *Serratia spp.* | | | | | | | | | | |
| piperacillin/tazobactam | 313 | 6.4 | 141 | 8.5 | 172 | 4.7 | 164 | 5.5 | 149 | 7.4 |
| cefoperazone/sulbactam^a^ | 312 | 16.0 | 141 | 12.8 | 171 | 18.7 | 163 | 11.7 | 149 | 20.8 |
| ceftazidime/avibactam | 83 | 0.0 | 51 | 0.0 | 32 | 0.0 | 52 | 0.0 | 31 | 0.0 |
| ceftazidime | 311 | 9.6 | 140 | 15.7 | 171 | 4.7 | 163 | 9.2 | 148 | 10.1 |
| ceftriaxone | 312 | 28.8 | 141 | 29.8 | 171 | 28.1 | 163 | 17.8 | 149 | 40.9 |
| cefepime | 313 | 18.5 | 141 | 15.6 | 172 | 20.9 | 164 | 11.6 | 149 | 26.2 |
| moxalactam | 312 | 3.2 | 141 | 4.3 | 171 | 2.3 | 163 | 3.7 | 149 | 2.7 |
| aztreonam | 313 | 16.0 | 141 | 22.0 | 172 | 11.0 | 164 | 12.2 | 149 | 20.1 |
| ertapenem | 169 | 5.3 | 72 | 11.1 | 97 | 1.0 | 98 | 7.1 | 71 | 2.8 |
| imipenem | 313 | 7.0 | 141 | 12.1 | 172 | 2.9 | 164 | 7.3 | 149 | 6.7 |
| meropenem | 313 | 5.1 | 141 | 9.2 | 172 | 1.7 | 164 | 6.1 | 149 | 4.0 |
| amikacin | 313 | 1.0 | 141 | 0.7 | 172 | 1.2 | 164 | 1.2 | 149 | 0.7 |
| gentamicin | 313 | 20.1 | 141 | 15.6 | 172 | 23.8 | 164 | 8.5 | 149 | 32.9 |
| ciprofloxacin | 312 | 30.4 | 140 | 30.0 | 172 | 30.8 | 164 | 22.0 | 148 | 39.9 |
| levofloxacin | 313 | 25.9 | 141 | 24.8 | 172 | 26.7 | 164 | 17.7 | 149 | 34.9 |
| trimethoprim/sulfamethoxazole | 313 | 6.1 | 141 | 6.4 | 172 | 5.8 | 164 | 4.3 | 149 | 8.1 |
| fosfomycin | 312 | 0.6 | 141 | 0.7 | 171 | 0.6 | 163 | 1.2 | 149 | 0.0 |
| tigecycline | 312 | 8.0 | 141 | 7.8 | 171 | 8.2 | 163 | 9.8 | 149 | 6.0 |
| *Salmonella spp.* | | | | | | | | | | |
| amoxicillin | 384 | 57.6 | 188 | 60.6 | 196 | 54.6 | 207 | 58.0 | 177 | 57.1 |
| amoxicillin/clavulanic acid | 384 | 7.6 | 188 | 9.0 | 196 | 6.1 | 207 | 6.7 | 177 | 8.5 |
| piperacillin/tazobactam | 384 | 2.1 | 188 | 3.7 | 196 | 0.5 | 207 | 2.4 | 177 | 1.7 |
| cefoperazone/sulbactam^a^ | 384 | 1.6 | 188 | 2.7 | 196 | 0.5 | 207 | 1.4 | 177 | 1.7 |
| ceftazidime/avibactam | 117 | 0.0 | 56 | 0 | 61 | 0 | 75 | 0.0 | 42 | 0 |
| ceftazidime | 384 | 9.9 | 188 | 11.7 | 196 | 8.2 | 207 | 8.7 | 177 | 11.3 |
| ceftriaxone | 384 | 10.9 | 188 | 12.8 | 196 | 9.2 | 207 | 9.2 | 177 | 13.0 |
| cefepime | 384 | 5.7 | 188 | 6.9 | 196 | 4.6 | 207 | 4.8 | 177 | 6.8 |
| moxalactam | 384 | 1.0 | 188 | 1.6 | 196 | 0.5 | 207 | 1.0 | 177 | 1.1 |
| aztreonam | 384 | 6.2 | 188 | 7.4 | 196 | 5.1 | 207 | 6.3 | 177 | 6.2 |
| ertapenem | 201 | 0.5 | 94 | 1.1 | 107 | 0 | 109 | 0.0 | 92 | 1.1 |
| imipenem | 384 | 0.3 | 188 | 0.5 | 196 | 0 | 207 | 0.0 | 177 | 0.6 |
| meropenem | 384 | 3.6 | 188 | 2.7 | 196 | 4.6 | 207 | 3.9 | 177 | 3.4 |
| amikacin | 384 | 1.0 | 188 | 1.1 | 196 | 1.0 | 207 | 1.0 | 177 | 1.1 |
| gentamicin | 384 | 7.3 | 188 | 7.4 | 196 | 7.1 | 207 | 9.7 | 177 | 4.5 |
| ciprofloxacin | 384 | 18.0 | 188 | 19.7 | 196 | 16.3 | 207 | 21.7 | 177 | 13.6 |
| levofloxacin | 384 | 8.6 | 188 | 10.1 | 196 | 7.1 | 207 | 11.6 | 177 | 5.1 |
| trimethoprim/sulfamethoxazole | 384 | 13.8 | 188 | 13.3 | 196 | 14.3 | 207 | 14.5 | 177 | 13.0 |
| fosfomycin | 384 | 0.5 | 188 | 0.5 | 196 | 0.5 | 207 | 0.5 | 177 | 0.6 |
| polymyxin B | 384 | 38.3 | 188 | 38.3 | 196 | 38.3 | 207 | 36.7 | 177 | 40.1 |
| tigecycline | 384 | 1.3 | 188 | 2.7 | 196 | 0.0 | 207 | 2.4 | 177 | 0 |
| *A. baumannii* | | | | | | | | | | |
| CRAB | 1062 | 64.2 | 670 | 71.3 | 392 | 52.0 | 568 | 56.7 | 494 | 72.9 |
| piperacillin/tazobactam | 1062 | 55.9 | 670 | 61.6 | 392 | 46.2 | 568 | 53.5 | 494 | 58.7 |
| cefoperazone/sulbactam^a^ | 1062 | 65.6 | 670 | 72.1 | 392 | 54.6 | 568 | 59.3 | 494 | 72.9 |
| ceftazidime | 1062 | 66.4 | 670 | 72.2 | 392 | 56.4 | 568 | 59.3 | 494 | 74.5 |
| ceftriaxone | 872 | 67.4 | 558 | 73.1 | 314 | 57.3 | 496 | 62.5 | 376 | 73.9 |
| cefepime | 1062 | 65.3 | 670 | 71.2 | 392 | 55.4 | 568 | 57.7 | 494 | 74.1 |
| imipenem | 1062 | 63.6 | 670 | 70.9 | 392 | 51.0 | 568 | 56.5 | 494 | 71.7 |
| meropenem | 1062 | 63.6 | 670 | 70.6 | 392 | 51.5 | 568 | 56.0 | 494 | 72.3 |
| amikacin | 1062 | 51.5 | 670 | 57.8 | 392 | 40.8 | 568 | 42.3 | 494 | 62.1 |
| gentamicin | 1062 | 60.4 | 670 | 64.6 | 392 | 53.1 | 568 | 51.9 | 494 | 70.0 |
| ciprofloxacin | 1062 | 65.2 | 670 | 72.5 | 392 | 52.6 | 568 | 58.1 | 494 | 73.3 |
| levofloxacin | 1062 | 58.6 | 670 | 65.2 | 392 | 47.2 | 568 | 54.6 | 494 | 63.2 |
| trimethoprim/sulfamethoxazole | 1062 | 53.8 | 670 | 59.0 | 392 | 44.9 | 568 | 49.8 | 494 | 58.3 |
| fosfomycin | 1062 | 6.7 | 670 | 5.2 | 392 | 9.2 | 568 | 7.2 | 494 | 6.1 |
| tigecycline | 1062 | 4.3 | 670 | 4.6 | 392 | 3.8 | 568 | 4.0 | 494 | 4.7 |
| *P. aeruginosa* | | | | | | | | | | |
| CRPA | 1044 | 20.1 | 609 | 25.5 | 435 | 12.6 | 574 | 25.3 | 470 | 13.8 |
| piperacillin/tazobactam | 1044 | 10.4 | 609 | 13.0 | 435 | 6.9 | 574 | 12.7 | 470 | 7.7 |
| cefoperazone-sulbactam^a^ | 1044 | 12.4 | 609 | 16.1 | 435 | 7.1 | 574 | 15.3 | 470 | 8.7 |
| ceftazidime/avibactam | 362 | 2.8 | 219 | 2.3 | 143 | 3.5 | 209 | 2.4 | 153 | 3.3 |
| ceftazidime | 1044 | 10.2 | 609 | 13.1 | 435 | 6.0 | 574 | 12.0 | 470 | 7.9 |
| ceftriaxone | 1044 | 7.8 | 609 | 10.5 | 435 | 3.9 | 574 | 8.7 | 470 | 6.6 |
| aztreonam | 1044 | 14.4 | 609 | 15.3 | 435 | 13.1 | 574 | 16.2 | 470 | 12.1 |
| imipenem | 1044 | 18.8 | 609 | 23.3 | 435 | 12.4 | 574 | 23.3 | 470 | 13.2 |
| meropenem | 1044 | 13.5 | 609 | 17.4 | 435 | 8.0 | 574 | 17.4 | 470 | 8.7 |
| amikacin | 1044 | 2.2 | 609 | 2.6 | 435 | 1.6 | 574 | 1.4 | 470 | 3.2 |
| gentamicin | 1044 | 5.7 | 609 | 5.7 | 435 | 5.5 | 574 | 4.9 | 470 | 6.6 |
| ciprofloxacin | 1044 | 18.2 | 609 | 21.5 | 435 | 13.6 | 574 | 22.6 | 470 | 12.8 |
| levofloxacin | 1044 | 17.2 | 609 | 19.7 | 435 | 13.8 | 574 | 21.4 | 470 | 12.1 |
| fosfomycin | 367 | 3.5 | 200 | 5.5 | 167 | 1.2 | 184 | 5.4 | 183 | 1.6 |
| polymyxin B | 1044 | 0.0 | 609 | 0 | 435 | 0 | 574 | 0 | 470 | 0 |
| tigecycline | 183 | 42.6 | 82 | 42.7 | 101 | 42.6 | 73 | 52.1 | 110 | 36.4 |

Abbreviation: MRSA, methicillin-resistant *S. aureus*; ESBL-EC, extended-spectrum β-lactamase-producing*E. coli*; ESBL-KP, extended-spectrum β-lactamase-producing *K. pneumonia*; CR-EC, carbapenem-resistant *E. coli*; CR-KP, carbapenem-resistant *K. pneumonia*; CR-AB, carbapenem-resistant *A. baumannii*; CR-PA, carbapenem-resistant *P. aeruginosa*.

^a^ Criteria as published by the CLSI[1] for cefoperazone used for cefoperazone-sulbactam.

Supplemental Table 3 The susceptibility and resistance of MRSA and MRCNS to antimicrobial agents

| Antibiotics | MRSA (2014-2019) | | | | | | MRCNS (2014-2019) | | | | | |
| --- | --- | --- | --- | --- | --- | --- | --- | --- | --- | --- | --- | --- |
|  | Number | MIC Range (μg/mL) | MIC_50_ | MIC_90_ | %R | %S | Number | MIC Range (μg/mL) | MIC_50_ | MIC_90_ | %R | %S |
| amikacin | 891 | 0.25 - 128 | 4 | 32 | 10.4 | 77.1 | 2020 | 0.06 - 256 | 4 | 16 | 6.3 | 88 |
| gentamicin | 715 | 0.06 - 32 | 1 | 32 | 26.0 | 73.0 | 1343 | 0.03 - 32 | 8 | 32 | 46.5 | 46.5 |
| rifampicin | 891 | 0.004 - 16 | 0.016 | 1 | 7.7 | 91.4 | 2020 | 0.004 - 16 | 0.016 | 1 | 8.7 | 90.6 |
| ciprofloxacin | 715 | 0.06 - 32 | 1 | 32 | 38.9 | 58.5 | 1343 | 0.03 - 32 | 8 | 32 | 56.7 | 36.7 |
| levofloxacin | 891 | 0.06 - 32 | 0.5 | 32 | 35.5 | 62.6 | 2021 | 0.015 - 64 | 4 | 32 | 57.3 | 38.5 |
| moxifloxacin | 891 | 0.015 - 32 | 0.25 | 16 | 35.6 | 60.4 | 2021 | 0.015 - 128 | 2 | 16 | 51.1 | 40.5 |
| trimethoprim/sulfamethoxazole | 891 | 0.015 - 16 | 0.064 | 0.5 | 4.9 | 95.1 | 2021 | 0.015 - 32 | 1 | 8 | 37.0 | 63.0 |
| clindamycin | 891 | 0.03 - 32 | 32 | 32 | 59.4 | 38.8 | 2021 | 0.03 - 64 | 0.25 | 32 | 43.4 | 52.5 |
| erythromycin | 891 | 0.25 - 32 | 32 | 32 | 81.9 | 13.4 | 2021 | 0.06 - 64 | 32 | 32 | 85.1 | 9.6 |
| daptomycin | 891 | 0.03 - 32 | 0.5 | 1 | 0 | 97.8 | 2021 | 0.03 - 8 | 0.5 | 1 | 0 | 99.7 |
| linezolid | 891 | 0.125 - 4 | 1 | 2 | 0 | 100 | 2021 | 0.06 - 32 | 1 | 2 | 1.7 | 98.3 |
| vancomycin | 891 | 0.25 - 2 | 1 | 2 | 0 | 100 | 2021 | 0.06 - 32 | 1 | 2 | 0 | 100 |
| teicoplanin | 715 | 0.06 - 8 | 0.5 | 2 | 0 | 100 | 1344 | 0.06 - 8 | 0.5 | 2 | 0 | 100 |
| tigecycline | 891 | 0.03 - 32 | 0.125 | 0.5 | 3.4 | 96.6 | 2021 | 0.015 - 4 | 0.25 | 0.5 | 7.9 | 92.1 |

Supplemental Table 4 The MDR prevalence of major pathogens to antimicrobial agents by hospital level and region economic development

|  | Tertiary hospitals | | Non-tertiary hospitals | | *p* | Developed Regions | | Developing Regions | | *p* |
| --- | --- | --- | --- | --- | --- | --- | --- | --- | --- | --- |
|  | Number | %MDR | Number | %MDR |  | Number | %MDR | Number | %MDR |  |
| MRSA | 1285 | 31.0 | 1558 | 31.6 | 0.759 | 1541 | 33.9 | 1302 | 28.3 | 0.002 |
| MDR-*S. aureu* | 1285 | 56.2 | 1558 | 53.9 | 0.241 | 1541 | 57.5 | 1032 | 65.5 | 0 |
| MDR-*E. faecium* | 592 | 93.8 | 358 | 91.1 | 0.156 | 524 | 92.9 | 426 | 92.5 | 0.378 |
| MDR-*E. faecalis* | 350 | 39.4 | 286 | 38.8 | 0.939 | 370 | 37.6 | 266 | 41.4 | 0.378 |
| MDR-*E. coli* | 4678 | 84.0 | 5266 | 78.9 | 0 | 5087 | 79.2 | 4857 | 83.6 | 0 |
| ESBL-*E. ccoli* | 4678 | 56.7 | 5266 | 50.4 | 0 | 5087 | 48.8 | 4857 | 58.2 | 0 |
| CR-*E. coli* | 4678 | 2.0 | 5266 | 1.0 | 0 | 5087 | 1.3 | 4857 | 1.6 | 0.229 |
| MDR-*K. pneumonia* | 2310 | 45.9 | 2068 | 27.6 | 0 | 2481 | 47.6 | 1897 | 49.6 | 0.19 |
| ESBL-*K. pneumonia* | 2310 | 36.6 | 2068 | 28.0 | 0 | 2481 | 29.6 | 1897 | 36.4 | 0 |
| CR-*K. pneumonia* | 2310 | 25.6 | 2068 | 10.2 | 0 | 2481 | 20.3 | 1897 | 15.7 | 0 |
| MDR-*A. baumannii* | 670 | 74.9 | 392 | 61.2 | 0 | 568 | 63.7 | 494 | 76.9 | 0 |
| CR-*A. baumannii* | 670 | 71.3 | 392 | 52.0 | 0 | 568 | 56.7 | 494 | 72.9 | 0 |
| MDR-*P. aeruginosa* | 609 | 25.3 | 435 | 20.2 | 0.067 | 574 | 26.7 | 470 | 18.9 | 0.004 |
| CR-*P. aeruginosa* | 609 | 25.5 | 435 | 12.6 | 0 | 574 | 25.3 | 470 | 13.8 | 0 |

Supplement Table 5 The susceptibility and resistance of other pathogens to antimicrobial agents

|  | All strains (204-2019) | | | | | |
| --- | --- | --- | --- | --- | --- | --- |
|  | Number | MIC Range (μg/mL) | MIC_50_ | MIC_90_ | %R | %S |
| *S. pneumoniae* | | | | | | |
| penicillin G(Non-meningitis) | 96 | 0.03 - 32 | 0.25 | 2 | 1.0 | 97.9 |
| penicillin G(Oral) | 96 | 0.03 - 32 | 0.25 | 2 | 25.0 | 36.5 |
| penicillin G (Meningitis) | 96 | 0.03 - 32 | 0.25 | 2 | 63.5 | 36.5 |
| cefuroxime (Oral) | 96 | 0.015 - 32 | 1 | 8 | 40.6 | 50.0 |
| cefuroxime (Parenteral) | 96 | 0.015 - 32 | 1 | 8 | 50.0 | 46.9 |
| ceftriaxone (Non-meningitis) | 96 | 0.015 - 32 | 0.5 | 2 | 8.3 | 81.2 |
| ceftriaxone (Meningitis) | 96 | 0.015 - 32 | 0.5 | 2 | 18.8 | 57.3 |
| cefepime (Non-meningitis) | 66 | 0.03 - 8 | 1 | 2 | 7.6 | 78.8 |
| cefepime (Meningitis) | 66 | 0.03 - 8 | 1 | 2 | 21.2 | 48.5 |
| meropenem | 66 | 0.015 - 1 | 0.25 | 0.5 | 4.5 | 83.3 |
| levofloxacin | 96 | 0.06 - 32 | 1 | 4 | 6.2 | 88.5 |
| moxifloxacin | 96 | 0.03 - 32 | 0.25 | 0.5 | 4.2 | 94.8 |
| trimethoprim/sulfamethoxazole | 96 | 0.015 - 8 | 1 | 8 | 35.4 | 47.9 |
| clindamycin | 96 | 0.03 - 32 | 16 | 32 | 85.4 | 13.5 |
| erythromycin | 96 | 0.015 - 32 | 16 | 32 | 89.6 | 10.4 |
| daptomycin | 68 | 0.03 – 0.25 | 0.25 | 0.5 | 0 | 100 |
| linezolid | 96 | 0.03 - 2 | 0.5 | 2 | 0 | 100 |
| vancomycin | 96 | 0.015 - 1 | 0.5 | 0.5 | 0 | 100 |
| teicoplanin | 38 | 0.015 - 0.5 | 0.064 | 0.125 | 0 | 100 |
| tetracycline | 96 | 0.06 - 32 | 16 | 32 | 74.0 | 19.8 |
| tigecycline | 68 | 0.03 - 0.5 | 0.064 | 0.25 | 0 | 72.1 |
| *Proteus spp.* | | | | | | |
| amoxicillin | 140 | 0.25 - 256 | 128 | 256 | 61.4 | 29.3 |
| amoxicillin/clavulanic acid | 142 | 0.25 - 256 | 8 | 128 | 20.4 | 65.5 |
| piperacillin/tazobactam | 147 | 0.25 - 128 | 0.25 | 4 | 0.7 | 98.0 |
| cefoperazone/sulbactam^a^ | 149 | 0.06 - 128 | 2 | 8 | 0.7 | 98.0 |
| ceftazidime/avibactam | 68 | 0.06 - 8 | 0.064 | 0.25 | 0 | 100 |
| cefazolin | 135 | 0.25 - 128 | 8 | 128 | 55.6 | 35.6 |
| cefuroxime | 135 | 0.25 - 128 | 16 | 128 | 49.6 | 48.9 |
| ceftazidime | 149 | 0.06 - 128 | 0.25 | 2 | 5.4 | 94.0 |
| ceftriaxone | 147 | 0.03 - 128 | 0.25 | 16 | 41.5 | 56.5 |
| cefepime | 149 | 0.03 - 64 | 0.25 | 4 | 4.7 | 81.2 |
| cefoxitin | 147 | 0.25 - 128 | 1 | 8 | 6.8 | 90.5 |
| moxalactam | 147 | 0.06 - 64 | 0.25 | 0.25 | 2.7 | 97.3 |
| aztreonam | 147 | 0.06 - 64 | 0.125 | 1 | 4.1 | 94.6 |
| ertapenem | 86 | 0.007 - 0.015 | 0.008 | 0.016 | 0 | 100 |
| imipenem | 149 | 0.03 - 32 | 0.5 | 1 | 1.3 | 90.6 |
| meropenem | 149 | 0.015 - 64 | 0.032 | 0.064 | 3.4 | 96.6 |
| amikacin | 149 | 0.25 - 128 | 2 | 16 | 4.7 | 92.6 |
| gentamicin | 149 | 0.25 - 128 | 2 | 128 | 32.2 | 54.4 |
| ciprofloxacin | 149 | 0.007 - 64 | 2 | 32 | 59.1 | 34.9 |
| levofloxacin | 149 | 0.015 - 64 | 1 | 16 | 45.6 | 45.0 |
| trimethoprim/sulfamethoxazole | 149 | 0.015 - 64 | 8 | 8 | 56.4 | 43.6 |
| fosfomycin | 147 | 0.06 - 1285 | 2 | 128 | 6.8 | 84.4 |
| *Citrobacter spp.* | | | | | | |
| piperacillin/tazobactam | 176 | 0.25 - 256 | 8 | 128 | 18.2 | 72.7 |
| cefoperazone/sulbactam^a^ | 176 | 0.06 - 128 | 8 | 128 | 16.5 | 71.6 |
| ceftazidime/avibactam | 65 | 0.125 - 32 | 0.5 | 32 | 16.9 | 83.1 |
| ceftazidime | 176 | 0.06 - 128 | 4 | 64 | 42.0 | 51.7 |
| ceftriaxone | 176 | 0.03 - 128 | 16 | 64 | 55.7 | 43.8 |
| cefepime | 176 | 0.03 - 128 | 0.25 | 64 | 21.0 | 66.5 |
| moxalactam | 176 | 0.06 - 128 | 0.25 | 128 | 14.8 | 77.8 |
| aztreonam | 176 | 0.03 - 128 | 4 | 64 | 36.9 | 55.7 |
| ertapenem | 90 | 0.007 - 32 | 0.016 | 4 | 16.7 | 81.1 |
| imipenem | 176 | 0.03 - 32 | 0.25 | 4 | 10.2 | 87.5 |
| meropenem | 176 | 0.015 - 32 | 0.016 | 4 | 10.2 | 87.5 |
| amikacin | 176 | 0.25 - 128 | 2 | 32 | 8.0 | 89.8 |
| gentamicin | 176 | 0.06 - 128 | 2 | 128 | 30.1 | 69.3 |
| ciprofloxacin | 176 | 0.007 - 128 | 0.5 | 32 | 39.2 | 46.6 |
| levofloxacin | 176 | 0.015 - 128 | 0.5 | 16 | 34.1 | 55.7 |
| trimethoprim/sulfamethoxazole | 176 | 0.015 - 16 | 0.125 | 8 | 35.8 | 64.2 |
| fosfomycin | 176 | 0.03 - 256 | 0.5 | 128 | 3.4 | 88.6 |
| polymyxin B | 176 | 0.125 - 32 | 1 | 2 | 5.7 | 94.3 |
| tigecycline | 176 | 0.06 - 16 | 0.25 | 1 | 1.7 | 97.2 |
| *Stenotrophomonasmaltophilia* | | | | | | |
| cefoperazone/sulbactam^a^ | 121 | 0.25 - 64 | 4 | 8 | 1.7 | 95.0 |
| ceftazidime | 241 | 0.06 - 128 | 4 | 32 | 10.8 | 82.2 |
| levofloxacin | 241 | 0.03 - 64 | 0.5 | 4 | 8.3 | 89.2 |
| trimethoprim/sulfamethoxazole | 241 | 0.015 - 16 | 0.25 | 4 | 18.3 | 81.7 |
| *Burkholderia spp.* | | | | | | |
| cefoperazone/sulbactam^a^ | 100 | 0.25 - 128 | 32 | 128 | 42 | 43 |
| ceftazidime | 189 | 0.06 - 128 | 4 | 32 | 14.3 | 76.7 |
| meropenem | 193 | 0.015 - 64 | 8 | 16 | 14 | 43.5 |
| levofloxacin | 193 | 0.03 - 64 | 2 | 32 | 34.7 | 52.8 |
| trimethoprim/sulfamethoxazole | 193 | 0.015 - 64 | 0.5 | 8 | 24.4 | 75.6 |

^a^ Criteria as published by the CLSI[1] for cefoperazone used for cefoperazone-sulbactam.

Supplemental Table 6 The susceptibility and resistance of ESBL+, ESBL- and CR-*E. coli* to antimicrobial agents

| Antibiotic | ESBL+*E. coli* (2014-2019) | | | | | | ESBL-*E. coli* (2014-2019) | | | | | | | CR-*E. coli* (2014-2019) | | | | | |
| --- | --- | --- | --- | --- | --- | --- | --- | --- | --- | --- | --- | --- | --- | --- | --- | --- | --- | --- | --- |
|  | Number | MIC Range (μg/mL) | MIC_50_ | MIC_90_ | %R | %S | Number | MIC Range (μg/mL) | MIC_50_ | MIC_90_ | %R | %S | Number | | MIC Range (μg/mL) | MIC_50_ | MIC_90_ | %R | %S |
| amoxicillin | - | - | - | - | - | - | 4638 | 0.125 - 512 | 128 | 256 | 60.8 | 34.4 | - | | - | - | - | - | - |
| amoxicillin/clavulanic acid | 5306 | 0.25 - 256 | 16 | 256 | 47.0 | 28.9 | 4638 | 0.06 - 256 | 8 | 128 | 30.5 | 54.1 | - | | - | - | - | - | - |
| piperacillin/tazobactam | 5306 | 0.06 - 256 | 4 | 128 | 10.8 | 80.7 | 4638 | 0.06 - 256 | 2 | 8 | 3.7 | 92.8 | - | | - | - | - | - | - |
| cefoperazone/sulbactam^a^ | 5306 | 0.06 - 256 | 16 | 64 | 13.8 | 67.3 | 4638 | 0.06 - 128 | 0.5 | 8 | 2.4 | 95.9 | - | | - | - | - | - | - |
| ceftazidime/avibactam | 1879 | 0.03 - 32 | 0.5 | 2 | 1.8 | 98.2 | 1804 | 0.06 - 32 | 0.25 | 0.5 | 0.2 | 99.8 | 61 | | 0.5 - 32 | 32 | 32 | 60.7 | 39.3 |
| cefazolin | - | - | - | - | 97.7 | 1.9 | 4638 | 0.06 - 256 | 2 | 32 | 16.1 | 72.5 | - | | - | - | - | - | - |
| cefuroxime | - | - | - | - | 94.8 | 4.4 | 4638 | 0.06 - 256 | 4 | 16 | 9.6 | 84.0 | - | | - | - | - | - | - |
| ceftazidime | 5306 | 0.03 - 256 | 16 | 64 | 50.1 | 36.6 | 4638 | 0.03 - 256 | 0.125 | 1 | 4.5 | 93.6 | - | | - | - | - | - | - |
| ceftriaxone | 5306 | 0.03 - 256 | 64 | 64 | 97.2 | 2.6 | 4638 | 0.015 - 256 | 0.125 | 0.5 | 8.5 | 91.1 | - | | - | - | - | - | - |
| cefepime | 5306 | 0.015 - 128 | 8 | 64 | 37.0 | 26.0 | 4638 | 0.015 - 256 | 0.064 | 0.125 | 2.8 | 95.6 | - | | - | - | - | - | - |
| cefoxitin | 5306 | 0.125 - 256 | 8 | 64 | 18.5 | 69.0 | 4638 | 0.03 - 256 | 4 | 16 | 8.0 | 87.5 | - | | - | - | - | - | - |
| moxalactam | 5305 | 0.06 - 128 | 0.5 | 4 | 3.3 | 93.9 | 4638 | 0.03 - 128 | 0.25 | 1 | 1.6 | 97.5 | - | | - | - | - | - | - |
| aztreonam | 5306 | 0.03 - 256 | 32 | 64 | 63.6 | 22.1 | 4638 | 0.03 - 256 | 0.125 | 1 | 4.9 | 94.0 | 145 | | 0.06 - 256 | 64 | 128 | 76.6 | 19.3 |
| ertapenem | 2792 | 0.007 - 32 | 0.016 | 0.25 | 2.3 | 96.8 | 2703 | 0.007 - 32 | 0.016 | 0.032 | 0.8 | 99.0 | - | | - | - | - | - | - |
| imipenem | 5306 | 0.015 - 32 | 0.125 | 0.5 | 1.7 | 97.9 | 4638 | 0.015 - 32 | 0.125 | 0.25 | 1.0 | 98.7 | - | | - | - | - | - | - |
| meropenem | 5306 | 0.007 - 32 | 0.032 | 0.064 | 1.7 | 98.2 | 4638 | 0.007 - 32 | 0.016 | 0.064 | 1.0 | 98.9 | - | | - | - | - | - | - |
| amikacin | 5306 | 0.25 - 256 | 4 | 8 | 4.4 | 94.9 | 4638 | 0.25 - 256 | 4 | 8 | 1.2 | 98.7 | 145 | | 0.5 - 128 | 4 | 128 | 26.9 | 71.0 |
| gentamicin | 5306 | 0.03 - 256 | 4 | 128 | 46.6 | 51.1 | 4638 | 0.03 - 256 | 2 | 128 | 31.3 | 67.4 | 145 | | 0.125 - 256 | 64 | 128 | 64.8 | 32.4 |
| ciprofloxacin | 5306 | 0.007 - 256 | 32 | 32 | 79.6 | 14.0 | 4638 | 0.007 - 256 | 0.5 | 32 | 46.9 | 39.8 | 145 | | 0.015 - 128 | 32 | 64 | 94.5 | 5.5 |
| levofloxacin | 5306 | 0.015 - 128 | 16 | 32 | 70.8 | 21.2 | 4638 | 0.007 - 128 | 0.5 | 16 | 33.4 | 55.2 | 145 | | 0.03 - 128 | 32 | 32 | 90.3 | 6.2 |
| trimethoprim/sulfamethoxazole | 5306 | 0.0075 - 32 | 8 | 8 | 66.3 | 33.7 | 4638 | 0.0075 - 32 | 0.5 | 8 | 46.6 | 53.4 | 145 | | 0.015 - 16 | 8 | 8 | 80.7 | 19.3 |
| fosfomycin | 5306 | 0.03 - 256 | 0.5 | 32 | 2.9 | 92.0 | 4638 | 0.03 - 256 | 0.5 | 1 | 0.3 | 98.8 | 145 | | 0.06 - 256 | 4 | 256 | 12.4 | 71.7 |
| polymyxin B | 5306 | 0.03 - 32 | 0.5 | 2 | 2.6 | 97.4 | 4638 | 0.06 - 128 | 0.5 | 2 | 1.7 | 98.3 | 145 | | 0.125 - 32 | 0.5 | 2 | 4.1 | 95.9 |
| tigecycline | 5306 | 0.015 - 8 | 0.25 | 0.5 | 0.0 | 99.9 | 4638 | 0.015 - 2 | 0.25 | 0.5 | 0.0 | 100.0 | 145 | | 0.03 - 2 | 0.25 | 1 | 0.0 | 100.0 |

Abbreviation: ESBL+*E. coli*, extended-spectrum β-lactamase-producing *E. coli*; ESBL-*E. coli*, extended-spectrum β-lactamase-non-producing *E. coli*; CR-*E. coli*, carbapenem-resistant *E. coli*.

^a^ Criteria as published by the CLSI[1] for cefoperazone used for cefoperazone-sulbactam.

Supplemental table 7 The susceptibility and resistance of ESBL+, ESBL- and CR-*K. pneumoniae* to antimicrobial agents

| Antibiotic | ESBL+*K. pneumoniae* (2014-2019) | | | | | | ESBL-*K. pneumoniae* (2014-2019) | | | | | | CR-*K. pneumoniae* (2014-2019) | | | | | |
| --- | --- | --- | --- | --- | --- | --- | --- | --- | --- | --- | --- | --- | --- | --- | --- | --- | --- | --- |
|  | Number | MIC Range (μg/mL) | MIC_50_ | MIC_90_ | %R | %S | Number | MIC Range (μg/mL) | MIC_50_ | MIC_90_ | %R | %S | Number | MIC Range (μg/mL) | MIC_50_ | MIC_90_ | %R | %S |
| amoxicillin/clavulanic acid | 1208 | 0.25 - 256 | 64 | 128 | 66.4 | 17.9 | 2471 | 0.25 - 256 | 4 | 128 | 21.2 | 69.2 | - | - | - | - | - | - |
| cefoperazone/sulbactam^a^ | 1424 | 0.06 - 128 | 64 | 128 | 51.3 | 34.4 | 2954 | 0.06 - 128 | 0.25 | 128 | 12.1 | 86.7 | - | - | - | - | - | - |
| ceftazidime/avibactam | 623 | 0.06 - 32 | 2 | 8 | 4.0 | 96.0 | 1032 | 0.06 - 32 | 0.25 | 0.5 | 0.2 | 99.8 | 325 | 0.06 - 32 | 4 | 8 | 8.3 | 91.7 |
| piperacillin/tazobactam | 1424 | 0.06 - 256 | 64 | 128 | 43.7 | 41.9 | 2954 | 0.125 - 256 | 4 | 128 | 11.5 | 84.3 | - | - | - | - | - | - |
| cefazolin | - | - | - | - | - | - | 2954 | 0.125 - 256 | 1 | 128 | 18.4 | 76.0 | - | - | - | - | - | - |
| cefuroxime | - | - | - | - | - | - | 2954 | 0.125 - 256 | 4 | 128 | 18.1 | 78.5 | - | - | - | - | - | - |
| ceftazidime | 1424 | 0.03 - 128 | 32 | 64 | 70.3 | 20.6 | 2954 | 0.03 - 256 | 0.25 | 64 | 12.9 | 86.3 | - | - | - | - | - | - |
| ceftriaxone | 1424 | 0.03 - 256 | 64 | 64 | 94.2 | 5.1 | 2954 | 0.03 - 256 | 0.125 | 64 | 14.2 | 85.4 | - | - | - | - | - | - |
| cefepime | 1424 | 0.03 - 128 | 16 | 64 | 58.6 | 18.6 | 2954 | 0.015 - 256 | 0.064 | 32 | 11.8 | 87.1 | - | - | - | - | - | - |
| cefoxitin | 1424 | 0.25 - 128 | 16 | 128 | 49.6 | 43.7 | 2954 | 0.03 - 256 | 4 | 128 | 16.6 | 80.6 | - | - | - | - | - | - |
| moxalactam | 1424 | 0.06 - 128 | 2 | 128 | 31.8 | 63.8 | 2954 | 0.03 - 256 | 0.25 | 64 | 10.5 | 88.2 | - | - | - | - | - | - |
| aztreonam | 1424 | 0.03 - 256 | 64 | 64 | 78.2 | 16.6 | 2954 | 0.015 - 256 | 0.125 | 64 | 12.8 | 86.5 | 801 | 0.03 - 256 | 64 | 64 | 94.5 | 5.4 |
| ertapenem | 863 | 0.007 - 32 | 0.25 | 32 | 42.9 | 55.0 | 1655 | 0.007 - 32 | 0.016 | 0.25 | 8.9 | 90.9 | - | - | - | - | - | - |
| imipenem | 1424 | 0.015 - 32 | 0.25 | 32 | 32.0 | 67.1 | 2954 | 0.015 - 128 | 0.25 | 8 | 10.6 | 88.6 | - | - | - | - | - | - |
| meropenem | 1424 | 0.007 - 32 | 0.064 | 32 | 32.2 | 67.1 | 2954 | 0.007 - 64 | 0.032 | 4 | 10.3 | 89.5 | - | - | - | - | - | - |
| amikacin | 1424 | 0.25 - 128 | 2 | 128 | 25.8 | 73.8 | 2954 | 0.125 - 256 | 2 | 4 | 7.8 | 92.1 | 801 | 0.25 - 256 | 128 | 128 | 63.5 | 36.0 |
| gentamicin | 1424 | 0.03 - 128 | 32 | 128 | 57.6 | 40.4 | 2954 | 0.03 - 256 | 1 | 64 | 11.9 | 87.8 | 801 | 0.03 - 256 | 128 | 128 | 76.8 | 22.3 |
| ciprofloxacin | 1424 | 0.007 - 256 | 32 | 32 | 84.4 | 12.0 | 2954 | 0.007 - 256 | 0.064 | 32 | 25.3 | 70.8 | 801 | 0.007 - 256 | 32 | 32 | 95.5 | 4.1 |
| levofloxacin | 1424 | 0.015 - 128 | 8 | 32 | 67.8 | 16.1 | 2954 | 0.015 - 128 | 0.064 | 32 | 17.8 | 74.9 | 801 | 0.015 - 128 | 32 | 32 | 93.1 | 4.2 |
| trimethoprim/sulfamethoxazole | 1424 | 0.015 - 32 | 8 | 8 | 71.3 | 28.7 | 2954 | 0.015 - 16 | 0.125 | 8 | 20.2 | 79.8 | 801 | 0.015 - 32 | 8 | 8 | 65.3 | 34.7 |
| fosfomycin | 1424 | 0.03 - 256 | 8 | 256 | 10.1 | 82.8 | 2954 | 0.125 - 256 | 4 | 32 | 4.1 | 92.9 | 801 | 0.5 - 256 | 32 | 256 | 25.6 | 61.8 |
| polymyxin B | 1424 | 0.125 - 128 | 0.5 | 2 | 3.8 | 96.2 | 2954 | 0.125 - 128 | 1 | 2 | 3.5 | 96.5 | 801 | 0.125 - 32 | 0.5 | 2 | 4.5 | 95.5 |
| tigecycline | 1424 | 0.03 - 16 | 0.5 | 1 | 0.5 | 98.7 | 2954 | 0.015 - 32 | 0.25 | 1 | 0.1 | 99.1 | 801 | 0.03 - 8 | 0.5 | 2 | 0.2 | 97.9 |

Abbreviation: ESBL+*K. pneumoniae*, extended-spectrum β-lactamase-producing *K. pneumoniae*; ESBL-*K. pneumoniae*, extended-spectrum β-lactamase-non-producing *K. pneumoniae*; CR-*K. pneumoniae*, carbapenem-resistant *K. pneumoniae*.

^a^ Criteria as published by the CLSI^[1]^ for cefoperazone used for cefoperazone-sulbactam.

Reference

1. I Clinical and Laboratory Standards Institute. *Performance standards for antimicrobial susceptibility testing: M100-s30. Wayne, PA, USA: CLSI;*. 2020.
